# Exploiting acquired vulnerability to develop novel treatments for cholangiocarcinoma

**DOI:** 10.1186/s12935-024-03548-2

**Published:** 2024-11-05

**Authors:** Sirayot Areewong, Orawan Suppramote, Sunisa Prasopporn, Siwanon Jirawatnotai

**Affiliations:** 1 Siriraj Center of Research Excellence (SiCORE) for Systems Pharmacology, Department of Pharmacology, Faculty of Medicine, Siriraj Hospital, Mahidol University, 2 Wanglang Rd., 11th Floor Srisavarindhira Building, Bangkok Noi, 10700 Bangkok, Thailand; 2 Princess Srisavangavadhana College of Medicine, Chulabhorn Royal Academy, 906 Kampangpetch 6 Rd., Talat Bang Khen, Lak Si, 10210 Bangkok, Thailand; 3 Faculty of Pharmacy, Silpakorn University, 6 Ratchamankanai Road., Phra Pathom Chedi Sub-district, Mueang District, 73000 Nakhon Pathom, Thailand

**Keywords:** Acquired vulnerability, Cancer drug resistance, Cholangiocarcinoma, Drug combination

## Abstract

Cholangiocarcinoma (CCA) presents a formidable therapeutic challenge due to its extensive heterogeneity and plasticity, which inevitably lead to acquired resistance to current treatments. However, recent evidence suggests that acquired drug resistance is associated with a fitness cost resulting from the myriad of acquired alterations under the selective pressure of the primary treatment. Consequently, CCA patients with acquired resistance are more susceptible to alternative therapies that are ineffective as monotherapies. This phenomenon, termed “acquired vulnerability,” has garnered significant interest in drug development, as the acquired alterations could potentially be exploited therapeutically. This review elucidates the modes of acquired vulnerability, methods for identifying and exploiting acquired vulnerabilities in cancer (particularly in CCA), and strategies to enhance the clinical efficacy of drug combinations by leveraging the principle of acquired vulnerability. Identifying acquired vulnerabilities may pave the way for novel drug combinations to effectively treat highly heterogeneous and adaptable malignancies such as CCA.

## Introduction

Cholangiocarcinoma (CCA), the second most common primary hepatic malignancy, is frequently diagnosed at an unresectable advanced stage, necessitating systemic chemotherapy as the recommended treatment [1, 2]. Despite suggestions for innovative treatments, such as targeted therapies for druggable genes [3–6], satisfactory results have yet to be demonstrated, underscoring the urgent need for novel therapeutic approaches.

**Fig. 1:**
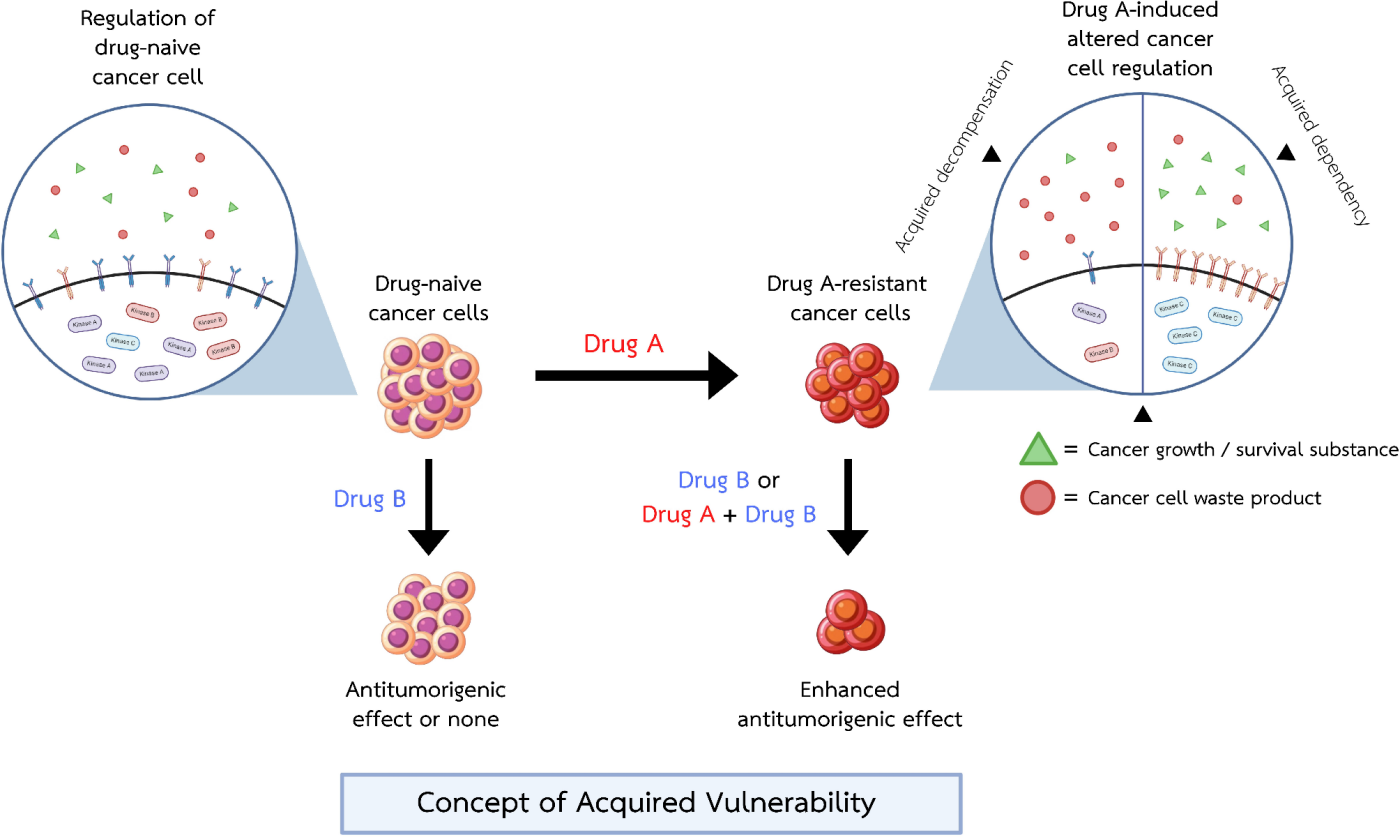
The concept of acquired vulnerability. Drug-naive cancer cells, with varying responses to drug B, can develop resistance to drug A upon exposure. This resistance is accompanied by targetable molecular alterations, known as acquired vulnerabilities. Drug B may exploit these vulnerabilities in two ways: (1) by targeting acquired dependencies on survival mechanisms induced by drug A or (2) by targeting acquired decompensations where cancer cells cannot fully mitigate drug A-induced damage

Under the pressure of treatment, cancer cells adapt to survive by reprofiling through numerous molecular pathways. Some altered pathways are retained and enriched due to their necessity for cancer cell survival under treatment pressure. The acquired changes that could, in turn, promote the sensitivity of resistant cancer to a particular treatment are known as “acquired vulnerability” (also referred to as “acquired sensitivity” or “collateral sensitivity”; Fig. 1). Acquired vulnerability has been widely explored as a potential solution for cancer that otherwise lacks effective therapy. This review classifies the types of acquired vulnerabilities demonstrated by recent studies and describes some of the procedures used to identify these vulnerabilities. Furthermore, the implementation of acquired vulnerability-based therapy, including combination strategies and assessments of drug synergism, is discussed. This review also mentions promising clinical applications of acquired vulnerability, and discusses the unique advantages and the shortcomings that still need further development.

## Cholangiocarcinoma: a formidable therapeutic challenge

CCA is a notoriously difficult-to-treat malignancy characterized by extensive heterogeneity and rapid development of resistance to standard therapies. Due to its silent clinical nature, CCA often manifests symptoms only in advanced stages, rendering surgical intervention impractical in most cases and leaving pharmacological therapies as the sole viable option [7]. However, pharmacological therapies for unresectable CCA have demonstrated limited efficacy. The combination of gemcitabine and cisplatin (GEM/CIS) serves as the standard first-line treatment, with the phase 3 ABC-02 clinical trial establishing an overall survival (OS) of 11.7 months and 81.4% tumor control. Efforts to improve OS have involved the addition of a third agent to the GEM/CIS combination, such as TS-1 (a prodrug of 5-fluorouracil) [8], nab-paclitaxel (an immune checkpoint inhibitor) [9], durvalumab [10], or pembrolizumab [11]. Despite promising results, the identification of a third agent that elicits substantial responses remains elusive.

Compared with active symptom control (ASC), the second-line therapy FOLFOX (fluorouracil, oxaliplatin, and leucovorin) can increase OS at 6 and 12 months by 15.1% and 14.5%, respectively. However, OS remains low: 6.2 months in the ASC plus FOLFOX group versus 5.3 months (4.1–5.8) in the ASC-alone group [12]. Utilizing capecitabine as adjuvant chemotherapy following surgery has also proven ineffective, with phase 3 clinical studies reporting only a modest increase in 5-year OS in the capecitabine group compared to the surgery-only group (8.64% and 3%, respectively), accompanied by reports of high-grade toxicity [13, 14]. Additional studies have revealed that the risk of tumor relapse in patients receiving various types of adjuvant therapy remains high, although the risk is slightly lower than that in observational patients [15–18].

Targeted therapy options for targeted therapy for CCA patients with aberrant FGFRs, IDH1/2 genes, namely pemigatinib (FGFR inhibitor) and ivosidenib (mutant IDH inhibitor), which cover only around 10–15% and 15–20% of the iCCA patients [19, 20]. Pemigatinib treatment had a complete response rate of about 2.8% with an ORR of 36% in CCA patients harboring *FGFR2* alterations [21]. The IDH-1 targeted therapy showed an ORR of 2%, a median OS of 10.8 months, and a median FPS of 2.7 months in a phase 3 trial [22]. Additionally, the results of other targeted therapies, such as inhibitors of EGFR, VEGF/VEGFR, HER2, etc. were modest [3, 6]. It is speculated that a plastic nature of CCA and its heterogeneity can be the culprit, which renders any targeted therapy that inhibits a single pathway ineffective.

Currently, immunotherapy for CCA treatment is still on the way of development. Novel CAR-T cell therapy is currently in a phase I clinical trial with a median progression-free survival of only 4 months [23]. Immune checkpoint inhibitor monotherapies, including pembrolizumab, nivolumab (in a phase 2 trial), and bintrafusp alfa (in a phase 1 trial), have shown similar median progression-free survival rates of approximately 2–4 months in advanced-stage patients [24–26]. In year, 2021, the development of Bintrafusp alfa has been terminated. However, novel drug regimens for CCA treatment are still urgently needed to benefit patients.

## Acquired vulnerability: a strategy to overcome drug-resistant cancers

Acquired vulnerability is a phenomenon in which cancer cells develop resistance to certain drugs while simultaneously becoming more susceptible or vulnerable to other treatments. This phenomenon was first mentioned in the early 1950s when Law observed that certain leukemia cell lines in mice resistant to the guanine analog 8-azaguanine were more sensitive to amethopterin than guanine analog-sensitive cell lines [27]. Concurrently, Szybalski and Bryson [28] discovered that *E. coli* strains resistant to a particular antibiotic could be up to a hundred-fold more sensitive to other antibiotics. The researchers termed this phenomenon “collateral sensitivity.” Subsequently, Julia, Kanai, and Charles applied the concept of collateral sensitivity to treat lymphoma cells in mice by combining 9-α-fluorohydrocortisone (9-AFH) and 5-fluorouracil (5-FU) [29]. They found that 5-FU specifically inhibited the incorporation of uracil-2-C^14^ into RNA in 9-AFH-resistant lymphoma cells. Combining the two drugs caused complete regression in mixed-type 9-AFH-sensitive and 9-AFH-resistant cell lines, with adaptive sensitivity to 5-FU in the 9-AFH-treated cells considered to indicate acquired vulnerability [30].

## Exploiting acquired vulnerability in various cancer types

**Fig. 2:**
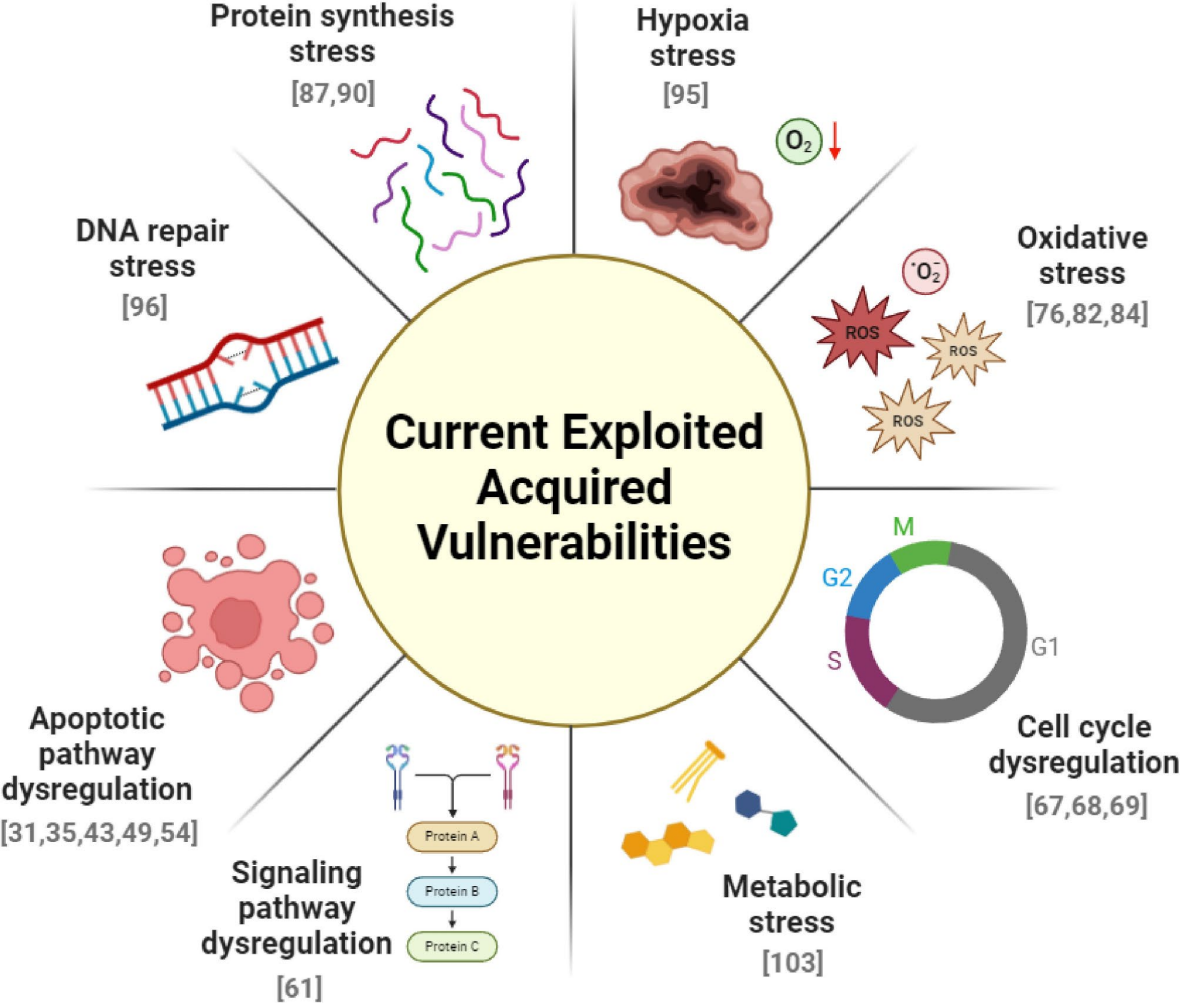
Exploited acquired vulnerabilities. This figure illustrates categories of acquired vulnerabilities previously targeted by secondary treatments, demonstrating superior outcomes in preclinical models

In recent years, a growing body of research has focused on harnessing the potential of acquired vulnerability across a diverse range of malignancies. These studies have focused on various aspects of the adaptive changes in cancer cells that occur after primary treatment or between successive treatments. We identified and summarized the acquired vulnerabilities reported thus far in several cancer types, classifying them into eight categories distinguished by the acquired pathway vulnerabilities for secondary treatments (Fig. 2). Our review emphasizes cell-autonomous acquired vulnerability and excludes non-cell-autonomous vulnerabilities, such as the reprofiling of immune evasion, the tumor microenvironment, angiogenesis, or epigenetic alterations.

### Apoptotic pathway dysregulation

Compared with their drug-naive counterparts, resistant cancer cells often exhibit altered pro- and anti-apoptotic pathway profiles. Several studies have demonstrated that primary treatment renders cancer cells vulnerable to apoptosis. However, anti-apoptotic signals are often reprofiled to compensate for this vulnerability and maintain the survival of resistant cancer cells despite pressure from primary treatment. Consequently, exploiting the altered apoptotic pathway under primary therapy might be a viable approach. Piggott et al. focused on tamoxifen resistance in estrogen receptor-positive/HER2-negative (ER+/HER2-) breast cancer. They found posttranslational downregulation of the c-FLIP protein, indicating that tamoxifen-resistant breast cancer cells may utilize other mechanisms to maintain survival and compensate for the decreased c-FLIP [31]. Oxidative stress caused by tamoxifen treatment activates the JNK/AP-1 axis [32] in the anti-oxidative response pathway [33], promoting c-FLIP degradation *via* the E3 ubiquitin ligase ITCH [38]. As a master anti-apoptotic regulator and resistance factor, c-FLIP suppresses apoptosis induced by tumor necrosis factor-α (TNF-α), Fas-L, and TNF-related apoptosis-inducing ligand (TRAIL). Researchers have demonstrated that tamoxifen-treated ER+/HER2- breast cancer cells are highly vulnerable to TRAIL receptor agonist treatment [31]. This treatment binds to death receptor 4/5, inducing Fas-associated death domain (FADD) adapter protein recruitment and subsequent caspase 8/10 recruitment to FADD [34]. Thus, the effect of the TRAIL receptor agonist is amplified in c-FLIP-downregulated cells.

Ruiz et al. demonstrated a significant downregulation of the CXCR2/Bcl-2 axis induced by taxane drugs in prostate cancer models [35]. The anti-apoptotic protein Bcl-2 inhibits cellular apoptosis by inactivating several caspases [36]. Despite the substantial downregulation of Bcl-2, prostate cancer cells may successfully develop drug resistance through other mechanisms, such as growth pathway stimulation or adaptation of the tumor microenvironment [37]. Consequently, Ruiz et al. hypothesized that increasing pro-apoptotic pressure in prostate cancer cells with low Bcl-2 expression would induce cell death, which they achieved by cisplatin treatment in their study [35]. Cisplatin activates pro-apoptotic pathways *via* DNA damage-regulated caspase activation [38]. The low amount of Bcl-2 protein in the prostate cancer cells was insufficient to counteract the strong pro-apoptotic signal induced by cisplatin treatment. Therefore, combining a taxane drug (docetaxel or cabazitaxel) with cisplatin resulted in better tumor suppression and longer survival in a mouse model [35]. Mechanistically, docetaxel impedes androgen receptor (AR) nuclear translocation and reduces AR signaling [39]. AR signaling is essential for maintaining the levels of CXCR and Bcl-2 proteins. Androgens have been shown to upregulate the expression of CXCR4, CXCR2, CXCR8, and, consequently, Bcl2 proteins through NF-κB-regulated transcription, increasing the metastatic capacity of prostate carcinoma cells [40–42].

The anti-apoptotic protein YAP is the main component of the Hippo signaling pathway. Iannelli et al. reported the acquired hyperactivation of YAP in docetaxel-resistant prostate cancer cell lines [43]. As YAP expression is promoted by the mevalonate pathway, Iannelli and colleagues targeted YAP by adding valproic acid (histone deacetylase inhibitor) and simvastatin (HMG-CoA reductase inhibitor) to the docetaxel regimen to inactivate the anti-apoptotic signal from YAP. Valproic acid and simvastatin synergistically induce AMPK activation, which facilitates the Ser127 phosphorylation and degradation of YAP. YAP degradation allows the expression of c-Myc, which promotes apoptosis in cancer cells [44]. Interestingly, acquired YAP activation post docetaxel resistance is correlated with activated ERK1/2 signaling [45], which can suppress PrLZ degradation [46], allowing PrLZ to decrease AMPK phosphorylation and activation. This results in the loss of AMPK-mediated negative feedback on HMG-CoA reductase, leading to uncontrollable YAP activation [47].

Inhibitors of apoptosis proteins (IAPs) negatively regulate pro-apoptotic pathways *via* direct inhibition of active caspases [48]. Dysregulation of IAPs can therefore be a target for secondary treatments. These proteins are often upregulated after resistance to primary treatment is acquired. For instance, Runckel et al. reported increased expression of several IAPs in rituximab-resistant B-cell lymphoma models [49], with activation of the mitogen-activated protein kinase (MAPK) and PI3K/AKT pathways suggested as the causes. Upon early-phase rituximab treatment, Bcl-2 family proteins are downregulated in leukemia cell lines due to preferential killing of CD20 + cells, causing decreased IL-10 production, inhibited STAT3 activation, and reduced interaction with the Bcl-2 promoter [50]. However, resistance to rituximab or persistent exposure to the medication results in rebound overexpression of Bcl-2 proteins [51], which is believed to be a result of hyperactivated MAPK and PI3K/AKT/mTOR signaling pathways [52, 53]. Bcl-2 overexpression leads to downstream IAP activation. Accordingly, rituximab-resistant cancer cells are hypersensitive to LCL-161 (IAP inhibitor).

Our laboratory detected the upregulation of cIAP2 in GEM/CIS-resistant CCA cell lines [54]. We targeted the altered cIAP2 expression using LCL-161, a SMAC mimetic [55]. LCL-161 strongly binds to the BIR3 domain of cIAP1 and cIAP2, causing their ubiquitination and proteasome degradation. This mechanism explains the pro-apoptotic role of LCL-161 in GEM/CIS-resistant CCA. GEM/CIS induces NF-κB upregulation, promoting cIAP2 overexpression at the transcriptional level. Although all three IAP proteins (cIAP1, cIAP2, and XIAP) can serve as protective mechanisms against DNA damage [56], we found that cIAP2 was the main IAP responsible for GEM/CIS resistance in CCA [54, 57–59]. The synergistic effects of GEM/CIS and IAP inhibitors were validated in patient-derived organoids and in vivo mouse xenografts.

### Growth signaling pathway dysregulation

Several studies have investigated drug resistance-induced vulnerabilities in growth signaling pathways, revealing potential targets for intervention. Acquired growth signaling vulnerabilities do not follow the traditional pathway bypass mechanisms, in which alternative receptor tyrosine kinases or MAPK members reactivate intracellular signals suppressed by primary treatments. Typically, primary treatments target oncogenic proteins such as receptor tyrosine kinases and MAPK proteins, but bypassing of these proteins occurs through alternative pathways that maintain downstream signaling despite inhibition [60]. However, acquired growth signaling vulnerability is not due to the inhibition of known alternative or related growth signaling pathways.

A well-known example is the recently approved triplet therapy for ER+/HER2- breast cancer, which includes a CDK4/6 inhibitor, a PI3K/AKT/mTOR inhibitor, and endocrine therapy. The clinical application of these therapies is based on the concept of acquired vulnerability. O’Brien et al. reported an increased dependency on the PI3K/AKT/mTOR pathway as an escape mechanism following resistance to CDK4/6 inhibition in ER+/HER2- breast cancer models. This dependency increases the susceptibility of cancer to PI3K/AKT/mTOR pathway inhibition. Notably, some resistant breast cancer patients lack mutations in the PI3K/AKT/mTOR pathway, indicating that nongenetic reprofiling and hyperactivation of the PI3K/AKT/mTOR pathway play significant roles [61].

The p110α-selective PI3K inhibitor, alpelisib, disrupts phosphorylation of the pro-apoptotic protein Bad and post-transcriptionally activates caspase-3 and − 9 [62]. This results in the regression of CDK4/6-resistant models [63], suggesting that CDK4/6 and hormone deprivation therapy render breast cancer cells vulnerable to PI3K/AKT/mTOR pathway inhibition. Mechanistically, the inhibition of CDK4/6 upregulates CDK2, thereby activating the PI3K/AKT/mTOR pathway. Increased activity of cyclin E-CDK2 compensates for CDK4/6 inhibition [64, 65], and CDK2 alone can activate AKT through S477 and T479 phosphorylation [66]. Exploiting this PI3K vulnerability has proven to be highly effective, making triple combination therapy (a CDK4/6 inhibitor, a PI3K inhibitor, and endocrine therapy) one of the most potent treatment regimens for breast cancer patients in clinical settings.

### Cell cycle dysregulation

Several studies have proposed exploiting acquired cell cycle deregulation as a therapeutic strategy [67–69]. Secondary treatment targets focus on cell cycle checkpoints. Nassar et al. investigated the loss of *CDKN2A* expression paired with *NRAS* mutation in *BRAF*-V600E-mutant melanoma resistant to BRAF/MEK inhibitors [67]. The *CDKN2A* gene encodes the p16^INK4A^ protein, which inhibits CDK4/6-cyclin D1 complexes, facilitating Rb-dependent cell cycle arrest at the G1 phase. Loss of *CDKN2A* abolishes this negative feedback loop, resulting in resistance to BRAF/MEK inhibition and dependency on cell proliferation *via* the CDK4/6-cyclin D1 complex. To exploit this vulnerability, CDK4/6 inhibitors are used in conjunction with MEK inhibition to address persistent activation of the RAS-MEK axis.

Inhibiting the RAS-MEK pathway also prevents the phosphorylation and inactivation of several pro-apoptotic proteins, including Bad, Mcl-1, Bim, and caspase-9, thereby inducing apoptosis [70]. A correlation has been shown between *NRAS* mutation and loss of *CDKN2A* expression through promoter hypermethylation [71], potentially explaining the loss of p16^INK4A^ in BRAF/MEK inhibitor-resistant melanoma with NRAS mutations.

Vulnerability exploitation may also target the G2/M phase. Wang et al. reported that B-cell lymphoma cells rely on the G2/M checkpoint to recover from chemotherapeutic agent-induced DNA damage. DNA-damaged lymphoma cells exhibit high levels of Wee1-dependent phosphorylated Y15 CDK1, preventing progression from the G2 to the M phase. This dependency on G2/M phase arrest renders chemotherapy-resistant lymphoma more susceptible to G2/M checkpoint disruption. Using a Wee1 inhibitor to break through the G2/M checkpoint can induce massive cell death in chemotherapy-treated lymphoma [68]. Wee1 is a critical protein that regulates G1/S arrest, allowing cells with DNA damage to recover [72]. Therefore, Wee1 inhibition forces cells with incomplete DNA synthesis to prematurely enter the M phase, leading to mitotic catastrophe, where interphase chromatin is forced to condense [73, 74].

In a subset of CCA cell lines, Song et al. reported that cyclin D1 overexpression is a major mechanism of acquired resistance to palbociclib (CDK4/6 inhibitor) in intrahepatic CCA cell lines [69]. To counteract this, mTOR inhibitors were used to inhibit overexpressed cyclin D1. This secondary treatment induced cyclin D1 downregulation, leading to cancer cell death associated with Bcl-2 downregulation and caspase-3 activation [75].

### Oxidative stress

Acquired dysregulation of reactive oxygen species (ROS) has been identified as a mechanism of resistance to cisplatin in non-small cell lung cancer (NSCLC). Cisplatin-resistant NSCLC cells exhibit decreased cellular ROS upon treatment. However, these cells regain sensitivity to cisplatin when ROS levels are elevated by eprenetapopt (APR-246), which is a small molecule that restores TP53 function and suppresses cellular ROS scavenging proteins such as SLC7A11 and NRF2 [76].

The role of the reduced form of nicotinamide adenine dinucleotide phosphate (NADPH) in oxidative stress is bidirectional. It acts as an antioxidant by serving as a cofactor for glutathione (GSH) reductase, facilitating the conversion of oxidized glutathione (GSSG) to reduced GSH. Conversely, NADPH serves as a substrate for producing superoxide anions and H_2_O_2_*via* NADPH oxidase [77]. Reduced levels of NADPH oxidase decrease intracellular ROS in NSCLC cells, facilitating cancer cell survival.

Under normal conditions, Keap1 binds to and degrades Nrf2, inhibiting its anti-oxidative effects. Cisplatin-induced oxidative stress disrupts this Keap1-Nrf2 complex, allowing Nrf2 to negatively regulate NADPH oxidase. Additionally, Nrf2 mediates GSH production through the Nrf2/SLC7A11/GSH axis [78–80]. Loss of the Scribble protein, which protects NADPH oxidase 2 from proteasomal degradation, has also been observed in cisplatin-resistant NSCLC [81]. These mechanisms collectively reduce ROS levels in resistant cancer cells, conferring cisplatin resistance and increasing the vulnerability of these cells to APR-246-mediated Nrf2 degradation.

Increased oxidative stress has been linked to BRAF inhibition in melanoma with the BRAF-V600E mutation. Data indicate that following BRAF inhibition, oxidative phosphorylation (OXPHOS) is activated, leading to high levels of oxidative stress in melanoma cells [82, 83]. Inhibiting the BRAF-MEK pathway in melanoma promotes a shift from lactate-producing glycolysis to OXPHOS for ATP production. Under BRAF inhibitor treatment, BRAF-V600E melanoma cells become dependent on OXPHOS for survival. This acquired dependency can be exploited, as demonstrated by the hypersensitivity of BRAF-V600E melanoma cells to OXPHOS inhibitors such as 2,4-dinitrophenol, oligomycin A [83], and the ROS inducer elesclomol [82].

This bioenergetic switch is specific to melanoma and is mediated by the melanocyte lineage factor MITF. The activated BRAF-MEK pathway suppresses MITF expression in melanocytes. When BRAF-MEK inhibition occurs, MITF suppression is relieved, allowing elevated levels of MITF to promote PGC1α-mediated mitochondrial biogenesis and OXPHOS in melanoma [83].

Wang et al. used MAPK reactivation-mediated ROS elevation to kill melanoma cells by further increasing ROS production *via* the histone deacetylase inhibitor vorinostat [84]. Epigenetically, histone deacetylase inhibition suppresses the expression of the antioxidant gene SLC7A11. The SLC7A11/xCT transporter facilitates the intracellular transport of extracellular cystine, which is subsequently converted into cysteine, a rate-limiting precursor in GSH synthesis [85]. A reduction in SLC7A11 expression results in decreased GSH production, lowering the anti-ROS defenses of cancer cells. The ensuing insufficient redox compensation leads to apoptosis or ferroptosis in resistant cancer cells [86].

### Protein synthesis stress

The dependency on protein synthesis pathways has emerged as a survival mechanism for drug-resistant cancer cells. For instance, Yang et al. highlighted the acquired vulnerability of lung adenocarcinoma cells that are resistant to either pemetrexed or MEK inhibitors. These *KRAS*-mutant cells exhibit a strong dependency on endoplasmic reticulum stress signaling, making them selectively susceptible to inhibition of HSP90, the receptor tyrosine kinase AXL, eukaryotic translation initiation factor 4E (eIF4E), and the unfolded protein response (UPR). MEK inhibitor resistance enables *KRAS*-mutant lung cancer cells to bypass canonical KRAS effectors. However, hyperactive AXL/eIF4E signaling, increased endoplasmic reticulum protein turnover, and adaptive activation of the endoplasmic reticulum stress-relief UPR survival pathway are maintained by HSP90.

These drug-resistant lung adenocarcinoma cells are hypersensitive to HSP90 inhibitors, such as onalespib, luminespib, and ganetespib. Combining the HSP90 inhibitor onalespib with trametinib (MEK inhibitor) markedly enhanced the inhibitory effect of trametinib in preclinical patient-derived xenograft (PDX) models [87]. The cellular stress induced by these treatments disrupts protein folding within the endoplasmic reticulum lumen, leading to the accumulation of misfolded proteins and consequent endoplasmic reticulum stress. The UPR is activated in response to alleviate this stress [88]. The UPR comprises several pathways that facilitate the clearance of unfolded proteins. As an upstream regulator of the UPR, HSP90 inhibition prevents resistant cells from relieving endoplasmic reticulum stress, leading to Bcl2-mediated apoptosis [89].

Our laboratory revealed a significant increase in the dependency on the RPL29-induced survival pathway in CCA models resistant to CDK4/6 inhibitors [90]. Consequently, targeting RPL29 upregulation is a promising strategy for eliminating CDK4/6 inhibitor-resistant CCA. Oxaliplatin can reverse RPL29 upregulation. This anti-ribosome biogenesis drug is known to disrupt nucleolar components and Pol I function [91] as well as shorten the half-life of the RPL29 protein [90].

The mechanism by which CDK4/6 inhibition leads to RPL29 upregulation remains unclear, particularly in resistant cells. One possible explanation is that the activated PI3K-AKT-mTOR pathway in resistant clones causes collateral overproduction of ribosomal proteins [92]. This overproduction results in ribosomal biogenesis stress, leading to the translocation of RPL5 and RPL11 from the cytoplasm to the nucleus. These proteins bind to and inhibit the function of MDM2 in the nucleus, enabling TP53 activation and cancer cell apoptosis.

### Hypoxic stress

Hypoxic tumor microenvironments are common in solid tumors due to rapid tumor growth. Excessive growth limits oxygen access from the vasculature, leading to tumor hypoxia. In response, hypoxia-inducible factors (HIFs), particularly the oxygen-sensitive HIF1-α subunit, promote tumor metabolism through glycolysis [93, 94].

Chen et al. discovered that cisplatin resistance induces intracellular hypoxia and HIF1-α upregulation in several cancer cell lines, enhancing oxygen diffusion around the cells. This acquired intracellular hypoxia was exploited to target drug-resistant cells using an innovative liposome containing a hypoxia-activatable pro-cisplatin drug [95].

### DNA repair stress

Kim et al. identified an acquired dependency on the CHK1/ATR signaling pathway for DNA repair in ovarian cancer cells resistant to poly (ADP-ribose) polymerase (PARP) inhibitors, irrespective of *BRCA1/2* mutational status [96]. This dependency is consistent across various mechanisms of PARP inhibitor resistance. Crucially, this reliance on the CHK1/ATR pathway can be exploited by using an ATR inhibitor. The loss of PARP function impairs single-strand break repair, leading to double-strand breaks.

Following the occurrence of double-strand breaks, ATR activates CHK1, enhancing the capacity of cancer cells for homologous recombination-mediated DNA repair through CHK1-mediated phosphorylation and recruitment of RAD51. RAD51 forms filaments on single-stranded DNA at damage sites, increasing DNA repair efficiency through homology searches and strand exchange [97]. ATR inhibition suppresses the PARP inhibitor-induced upregulation of ATR/CHK1, resulting in the loss of the G2/M checkpoint and destabilization of the replication fork. Consequently, cancer cells with damaged DNA enter the M phase prematurely, leading to mitotic catastrophe and cell death [73].

### Metabolic stress

Metabolic reprogramming following drug resistance in cancer is an area of extensive study. Acquired alterations in metabolic pathways and potential therapeutic targets have been reported for various biochemical compounds, including monosaccharides [98], amino acids [99, 100], lipids [101], and nucleotides [102]. Targeting drug resistance-induced reliance on specific metabolic pathways is a promising therapeutic strategy. Several studies have demonstrated this concept. Chronic lymphocytic leukemia (CLL) cell lines resistant to ibrutinib, a Bruton’s tyrosine kinase inhibitor, show a marked dependency on NADPH accumulation to compensate for decreased GSH levels [103]. This acquired vulnerability could be targeted by inhibiting tricarboxylic acid (TCA) cycle-mediated NADPH synthesis.

Investigations have shown that activation of the PI3K/AKT/mTOR pathway in lymphoma cells treated with ibrutinib leads to NADPH upregulation and subsequent drug resistance [104, 105]. This activation is suggested to result from ibrutinib-induced downregulation of FOXO3a/PTEN, which are negative regulators of AKT [106]. AKT activation stimulates mTORC1, promoting preferential glutamine consumption in the TCA cycle [107]. Limited substrate input leads to decreased GSH synthesis and low GSH levels in resistant CLL cells. Resistant CLL cells produce NADPH through glutamate catabolism and the TCA cycle, with isocitrate dehydrogenase converting glutamate to α-ketoglutarate, generating NADPH. Additionally, malic enzymes convert malate to pyruvate, contributing to the NADPH pool.

This vulnerability can be exploited by using etomoxir to inhibit fatty acid oxidation through blockade of the enzyme carnitine palmitoyltransferase 1. Inhibition of this enzyme disrupts the TCA cycle by preventing fatty acyl CoA, a precursor of β-oxidation, from entering the mitochondria, which decreases malate availability and reduces NADPH synthesis from malate to pyruvate [108]. Consequently, oxidative stress remains uncompensated, leading to oxidative stress-mediated cancer cell death [86].

## Considerations for acquired vulnerability as a means for cancer therapy

Acquired vulnerability presents a promising approach for cancer therapy. The ability to predict, prevent, and overcome nongenetic, pathway-reprofiling drug resistance makes this paradigm uniquely appealing. Clinically, this principle has proven effective, as seen in the case of PIK3CA-AKT pathway vulnerability in CDK4/6 inhibitor-treated hormone receptor (HR) + breast cancer [61]. However, several factors require careful attention and consideration regarding the nature of acquired vulnerability in cancer. The following are some of the major issues.

### Acquired vulnerability as a functional phenotype

Acquired vulnerability relies on the nongenetic adaptation of cancer cells, leading to several implications. This type of adaptive vulnerability often does not involve genetic changes detectable by standard clinical methods. Instead, adaptations may involve altered gene expression, the redirection of survival pathways, posttranslational modifications, or protein activation through other mechanisms. Consequently, acquired vulnerability is considered a functional change rather than a genetic change.

Unlike drug resistance caused by secondary mutations in target molecules or pathways, which can be readily detected by simple polymerase chain reaction or DNA sequencing, pathway bypassing or reprofiling relies on nongenetic mechanisms. Consequently, it is challenging to determine whether such adaptation has occurred in cancer cells. Many publications on acquired vulnerability have not identified specific biomarkers for this phenomenon. Only a few studies have proposed biomarkers that can be conveniently tested in clinical settings. For instance, apoptosis vulnerability in CCA under GEM/CIS-based therapy was predicted by the transcriptional upregulation of IAPs [54].

Further complicating this issue, pathway reprofiling can result in subtle changes in unrelated pathways. For example, acquired vulnerability to metabolic modulating drugs occurs when CLL is treated with ibrutinib, a Bruton’s tyrosine kinase inhibitor [103]. A similar phenomenon is observed in the case of ribosome-dependent phenotypes in CCA treated with CDK4/6 inhibitors [90]. The identification of acquired vulnerabilities and associated markers is typically performed through screening large drug libraries.

A substantial pitfall in acquired vulnerability-based drug discovery is the lack of understanding of the underlying causes of this vulnerability. Few studies have offered mechanistic explanations. Mechanistic insight is needed to translate knowledge of acquired vulnerability into clinical practice.

### Validation of acquired vulnerability in patient-derived models

Acquired vulnerability can vary even within the same type of cancer. Therefore, clinical translation becomes challenging without a consensus biomarker or mechanistic insight to predict vulnerability. Validation of drug response using patient-derived models, such as patient-derived organoids (PDOs) or PDXs, is crucial for implementing this concept in clinical settings. PDOs and PDXs enable the growth of tumors from individual patients in an in vivo system, referred to as “avatar models.” These models are instrumental in personalized medicine approaches, offering predictive capacity for individual patients. By enabling personalized treatment, avatar models reduce the costs associated with nontargeted therapies and improve drug efficacy [109].

PDOs are especially suitable for integration into the acquired vulnerability-based drug discovery pipeline, as drug testing in PDOs derived from clinical samples can be performed within months of tissue retrieval [110]. This rapid turnaround allows for personalized validation of individual cases. Decisions on the types of drugs or regimens to test in PDOs can be guided by results from acquired vulnerability screenings in cell lines, helping clinicians refine their list of drug choices. However, the success rates of organoid cultures vary significantly across different tumor types [111, 112], limiting the applicability of validation to certain tumors. In tumor types where PDOs can be routinely cultured, drug candidates identified through acquired vulnerability screening are particularly valuable.

The application of PDXs in personalized therapy remains challenging due to the prolonged tumor latency (4–6 months) and variable engraftment rates across different cancers and hosts. Despite these challenges, PDX models are invaluable for biomarker identification in reprofiled tumors and are essential for evaluating standard drug treatments. Additionally, drug resistance models can be developed in PDX systems for individual patients prior to the onset of clinical drug resistance. This preemptive strategy facilitates the identification of acquired vulnerabilities in the patient avatar model.

### Acquired vulnerability-targeted therapy alone is generally inadequate

Several lines of evidence indicate that nongenetically acquired vulnerabilities under cancer therapy can develop rapidly and diminish upon withdrawal of the primary therapy [54, 90, 113]. Consequently, integrating acquired vulnerability-based therapy into the primary treatment regimen as a combination therapy—rather than as a monotherapy—is essential. For instance, in melanoma treated with a BRAF inhibitor, cancer cells became hypersensitive to small-molecule inhibitors of OXPHOS. The addition of the OXPHOS inhibitors enhanced the cytotoxicity against melanoma only when combined with the BRAF inhibitor vemurafenib [83].

Upregulation of RPL29 occurs exclusively in CCA cells under CDK4/6 inhibition. This phenomenon was observed in both drug-naive and CDK4/6 inhibitor-resistant cells [90]. Interestingly, the study revealed that RPL29 expression returned to normal levels after the discontinuation of CDK4/6 inhibitors during a drug holiday. Consequently, continuous CDK4/6 inhibition was necessary to exploit the vulnerability of CCA cells to oxaliplatin, a ribosome biogenesis inhibitor. The study also demonstrated that combining CDK4/6 inhibitors with oxaliplatin was more effective than sequential therapy with these drugs. Thus, combination therapy may be necessary to capitalize on the adaptability of cancer cells.

### Proposed workflow for acquired vulnerability-based drug combination and drug scheduling

Table 1 summarizes the two main features that characterize acquired vulnerability-based drug discovery studies. First, acquired vulnerability arises from pathway reprofiling, which inevitably develops under primary or standard therapy. Second, secondary treatments targeting reprofiling can be combined with primary treatments to exploit acquired vulnerabilities. These combinations are anticipated to result in dramatic and lasting responses.

**Fig. 3:**
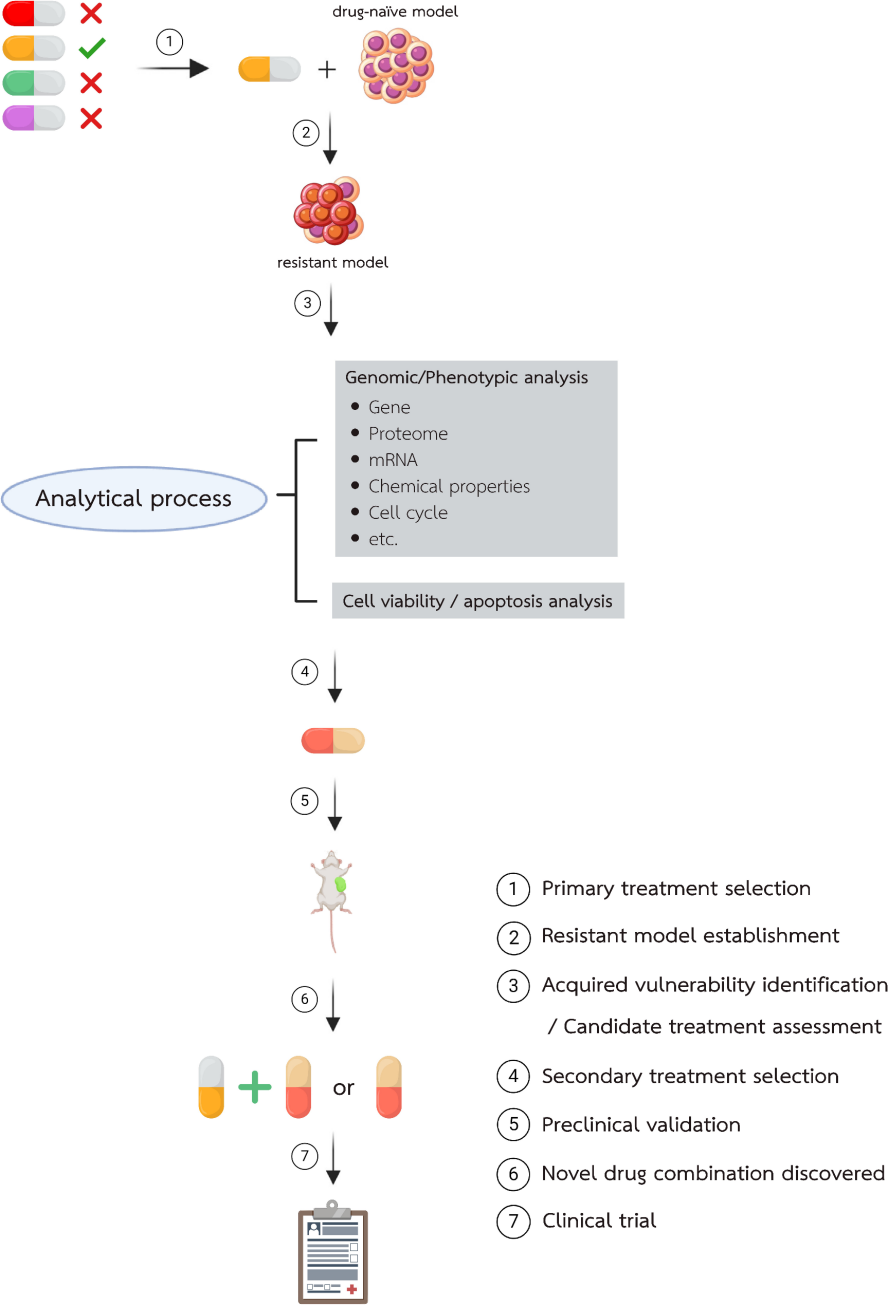
General workflow for acquired vulnerability-based drug discovery. Following primary treatment selection and administration in a cancer model, resistant clones are analyzed to (1) identify acquired vulnerabilities, (2) evaluate secondary treatment efficacy, and (3) elucidate the mechanisms of secondary treatment action on these vulnerabilities. Promising secondary treatment candidates are then tested in patient-derived organoids or animal models. If successful, a regimen incorporating secondary treatment is validated and proposed

The drug discovery pipeline for acquired vulnerability-based therapy can be generalized, as shown in Fig. 3. First, resistant clones are established under drugs of interest, such as GEM/CIS, 5-Fu, or FOLFOX standard therapy for CCA. The drugs could also be those used in nonstandard therapies, given that chemotherapies and targeted therapies have been shown to promote acquired vulnerability. Both monotherapies and combinations have been demonstrated to drive cancer reprofiling and acquired vulnerability.

Second, acquired vulnerability screening should be performed with a drug list covering as many pathways and biological processes as possible. This is because studies have often revealed acquired vulnerability from molecules/pathways that were previously unknown to connect to primary therapy. Our focus is on a double screening approach, in which drug-resistant (molecularly reprofiled) cancer cells are treated with individual drugs from our library in parallel with parental drug-naive cancer cells. Candidate drugs are those that specifically inhibit acquired drug-resistant cancer cells but have lower or no effect on drug-naive cancer cells. This simple scheme helps identify the new vulnerability (pathway or molecule) that drug-resistant cells gain from therapy-driven pathway reprofiling. Several alternative approaches may be applied to different goals. For example, an siRNA library can be used instead of a drug library [114], and libraries of noncancer drugs have also been used in previous studies [43].

Third, once the acquired vulnerability has been identified, different types of comparative analyses should be conducted to identify the relative changes in the cell before and after reprofiling. These analytical strategies aim to identify the reprofiled pathway/molecule that explains the acquired vulnerability. The analyses typically employ proteomic or transcriptomic-based methods, including reversed-phase protein array, mass spectrometry, or transcriptome analysis, with or without chromatin immunoprecipitation or assays for transposase-accessible chromatin with high-throughput sequencing. Protein/pathway-specific work-up plans are subsequently developed.

Fourth, identifying relevant biomarkers associated with reprofiling is equally important, considering CCA’s high intratumoral and interpatient heterogeneity. The acquired vulnerability caused by CCA molecular reprofiling is expected to vary from one patient to another and from one time point to the next, especially after any given therapy. Therefore, biomarker identification will help stratify patients in whom acquired vulnerability plays a role.

Last, validating the combined regimen or secondary treatment targeting acquired vulnerability in physiological models, such as PDOs or PDXs, will be highly useful before applying it to patients. This step allows the use of acquired vulnerability in an individualized manner and confirms the acquired vulnerability target when biomarkers for individuals may not be available.

### Drug combinations for acquired vulnerability therapy

Since almost all acquired vulnerability studies have favored using drug combinations rather than secondary treatment alone for attacking drug-resistant cancer, drug overdose is one limitation of using drug combinations.

Utilizing drug combinations based on acquired vulnerability has proven to be a powerful strategy for identifying synergistic combinations [115]. Drug synergism, a pharmacological phenomenon in which the combined effect of two or more drugs exceeds the sum of their individual effects, can reduce drug toxicity in patients by allowing for lower doses of each agent.

The combination index and the dose-reduction index are well-known parameters for evaluating the degree of drug synergism and can be calculated using various formulas [116–118]. The dose-reduction index value can be used to reduce the dose of each drug in the cocktail in clinical practice. Computer software, such as CompuSyn [54, 87] and CalcuSyn [43, 49, 67], can identify the combination index and dose-reduction index values of a given drug combination at given doses. These software programs can also determine the inhibitory concentration that results in an x% inhibition of cancer cells.

**Fig. 4:**
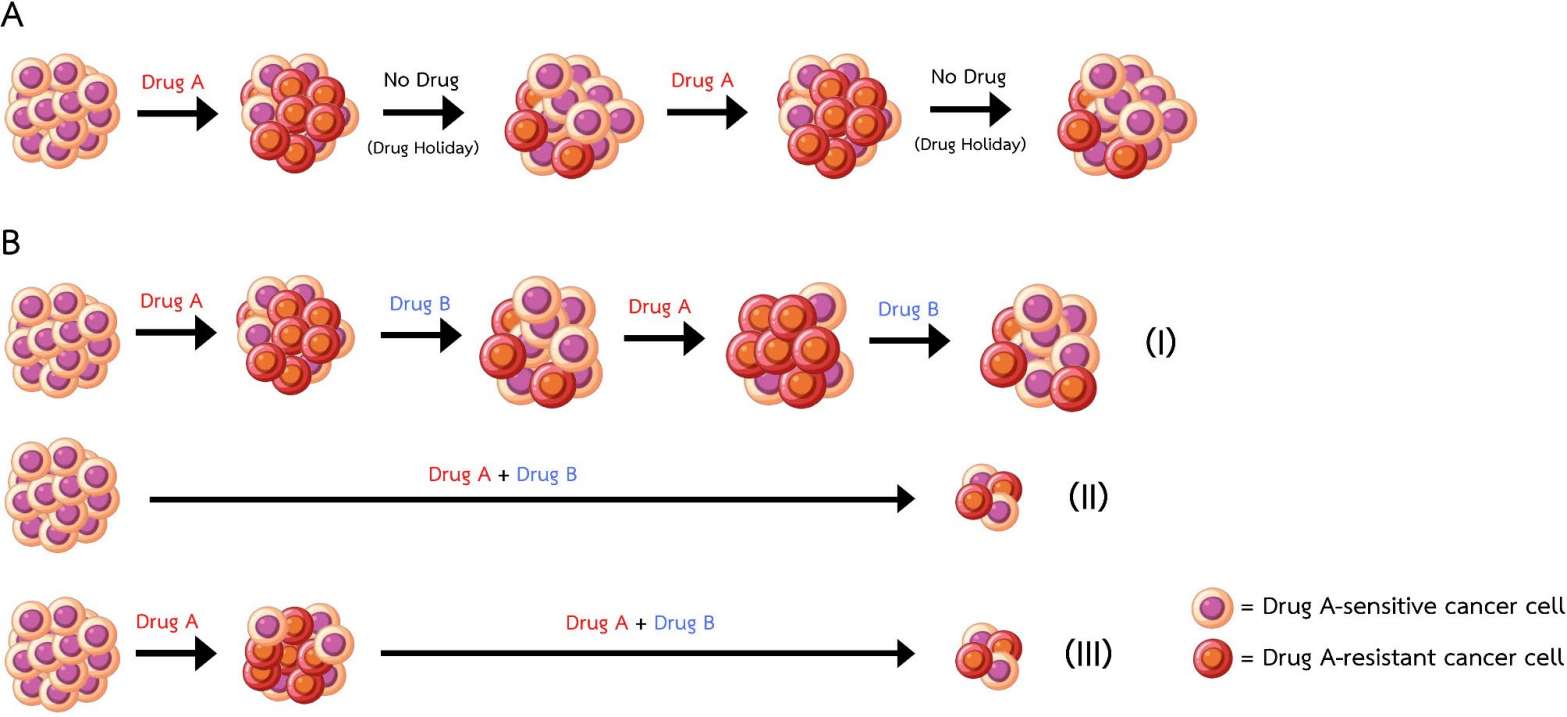
Regimen schedules in acquired vulnerability-based treatment research. **(A)** Intermittent dosing (monotherapy): Treatment is periodically interrupted after resistance develops, allowing some cells to revert to a nonresistant state. **(B)** Combination dosing: **(I)** Sequential dosing: Treatments are alternated after resistance develops to the latest treatment. **(II)** Preconcomitant dosing: Treatments are combined at the outset. **(III)** Postconcomitant dosing: Treatments are combined after resistance develops to the primary treatment

Some cancer-related treatment studies have utilized combination indices and dose-reduction indices to decrease the dose of each drug in 2-drug or 3-drug cocktails. Many cancer drug doses could be significantly reduced while still effectively eradicating various cancer cell types in vitro [119–123] and increasing survival and reducing tumor volume in vivo [124]. However, in recent acquired vulnerability-based studies, only the combination index values—but not the dose-reduction index values—for the drug combinations were determined. Furthermore, different regimen schedules were also shown to impact the efficacy of regimens (Fig. 4) [54, 90].

Applying acquired vulnerability screening to cancer drugs will increase the likelihood of identifying novel drug combinations, as it may reveal new targets previously unknown to contribute to resistance to primary therapy.

## Acquired vulnerability in CCA: an opportunity

The rapid development of resistance to standard chemotherapy, with most patients relapsing within 7 months [125], poses a significant challenge in CCA therapy. Furthermore, most CCA cases lack actionable mutations [126]. Although recurrent mutations frequently involve genes such as *TP53*,* KRAS*,* SMAD4*,* BRAF*,* MLL3*,* ARID1A*,* PBRM1*, and *BAP1* [127], these genes remain undruggable targets, as many of the genes function as tumor suppressors. While *KRAS G12C* inhibitors (such as sotorasib and adagrasib) show promise in NSCLC, the rarity of *KRAS G12C* in CCA (1.2–2.3%) [128–130] limits their utility. Clinical trials investigating various targeted therapies for CCA, including EGFR, HER2, VEGF, MET, CDK4/6, and pan-kinase inhibitors, have yielded disappointing results [131–138].

Emerging evidence suggests that tumorigenesis and treatment response in CCA are largely driven by dysregulated posttranscriptional signaling pathways [126, 139]. Consistent with this notion, CCA exhibits high plasticity and adaptability under current therapeutic regimens [140]. These findings imply that novel therapies should aim to exploit, rather than avoid, the rapid reprogramming of CCA cells.

Several lines of evidence indicate that CCA cells are vulnerable to therapeutic intervention. Recent data suggest that acquired metabolic stress is a potential target for CCA therapy. Compared to normal cholangiocytes, CCA cells exhibit numerous alterations in energy metabolism, including a reliance on the Warburg effect, suppressed TCA cycle activity, and increased pentose phosphate pathway flux, highlighting the importance of metabolic reprogramming in CCA carcinogenesis [141]. Additionally, evidence supports the role of stress from altered protein production and folding in CCA carcinogenesis. For example, the upregulation of HSP90 and its upstream regulator USP21 is essential for CCA cell growth and survival, with chaperone proteins facilitating HIF1α-mediated upregulation of key aerobic glycolysis proteins [142]. Furthermore, many CCA patients harbor defects in DNA repair mechanisms, as evidenced by frequent mutations in DNA repair genes such as *ARID1A*, *BAP1*, and *TP53*. These findings suggest that CCA carcinogenesis relies on a vulnerable DNA repair system. Currently approved PARP inhibitors may be able to target this vulnerability.

Our laboratory studies have demonstrated that targeting acquired vulnerabilities can effectively inhibit CCA. As CCA cells accumulate dysregulated pathways, they become primed for apoptosis, making them susceptible to BH3-mimetic drugs that disrupt the balance between pro- and anti-apoptotic factors. For example, several CCA cell lines acquired vulnerability to the apoptosis sensitizer LCL161 under standard GEM/CIS chemotherapy [54].

Although little is known about innate ribosomal stress in CCA, acquired stress can be generated by drug treatment. We have shown that CDK4/6 inhibition induces ribosomal biogenesis stress in CCA, leading to acquired vulnerability to ribosomal stressors such as oxaliplatin, phenanthriplatin, and actinomycin [90]. This finding warrants clinical translation, as a CDK4/6 inhibitor could potentially be added to the standard oxaliplatin-based CCA regimen.

Further research is needed to determine whether any of these dysregulated pathways are enhanced under CCA therapy and could be exploited clinically.

**Table 1:** Examples of acquired vulnerability-related research focusing on discovering new drug cocktails in various types of cancer

Cancer type	Resisted primary treatment	Interested acquired vulnerability	Selected secondary treatment	Causes of increased apoptosis in combination treatment	Results in preclinical model (approximate)	Reference
Lung cancer (KRAS-mutated)	Trametinib (MEK inhibitor)	Upregulation of the HSP90/AXL/eIF4E-regulated unfolded protein response	Onalespib (HSP90 inhibitor)	Inhibition of the acquired upregulation of HSP90/AXL/eIF4E-regulated unfolded protein response	**26-day tumor weight/volume (Patient-derived xenograft (PDX))** Vehicle – 1.1 g/18 cm² Trametinib – 1.05 g/15 cm² Onalespib – 0.98 g/13.5 cm² Combo – 0.38 g/5.9 cm²	[87]
Non-small cell lung cancer	Cisplatin (platinum-based chemotherapy)	Decrease NADPH oxidase-mediated ROS production	Eprenetapopt	Decrease GSH production through downregulation of NRF2/SLC7A11/GSH axis	**8-day treated tumor volume after cisplatin treatment at 650 mm² (**in vivo**)** Control – 15 cm² Eprenetapopt – 2.5 cm²	[76]
ER+/HER2- breast cancer	Ribociclib (CDK4/6 inhibitor) and fulvestrant (selective estrogen receptor degrader)	Upregulation of m-TOR signaling pathway	Alpelisib (p110α-selective PI3K inhibitor)	Inhibition of the acquired upregulation of m-TOR signaling pathway	**9-week treatment relapsing rate (**in vitro**)** Ribociclib – 100% Ribociclib + Fulvestrant – 71.4% Ribociclib + Fulvestrant + Alpelisib – 25%	[61]
	Tamoxifen (endocrine treatment)	Downregulation of c-FLIP anti-apoptotic protein	TRAIL receptor agonist	Increased activation of pro-apoptotic pathway	**Relative tumor volume of 4.5-week treatment after tamoxifen resistance (PDX)** Vehicle – 100% TRAIL receptor agonist – 25% Number of metastatic sites Vehicle – 25 sites TRAIL receptor agonist – 5 sites	[31]
Melanoma	Dabrafenib (BRAF inhibitor) and trametinib	Increase of ROS production	Vorinostat (Histone deacetylase inhibitor)	Further increase of ROS level through the inhibition of the ROS scavenging pathway	**Tumor volume of 40-day treatment after MAPK inhibitor resistance (**in vivo**)** Vehicle – 7 cm² BRAF inhibitor – 14 cm² Vorinostat – 2 cm²	[84]
	Vemurafenib (BRAF inhibitor) + Trametinib	Activation of cell-cycle pathway and reactivation of MAPK pathway	Palbociclib (CDK4/6 inhibitor) + Trametinib	Inhibition of the acquired activation of cell-cycle pathway and the reactivation of MAPK pathway	**Tumor volume of 30-day treatment of post-BRAF/MEK inhibitor resistant cancer (PDX)** Vehicle – 9.5 cm² Trametinib – 7.75 cm² Palbociclib – 7.15 cm² Combo – 3.2 cm²	[67]
	Vemurafenib	Increase oxidative phosphorylation-mediated ROS production	Elesclomol	Further increase the oxidative phosphorylation-mediated ROS production	**Tumor volume of 20-day treatment of post-BRAF inhibitor resistant cancer (PDX)** Vehicle – 20.5 cm² Vemurafinib – 14.5 cm² Elesclomol – 7.5 cm²	[82]
Hepatocellular carcinoma	Cisplatin	Increased intratumoral hypoxia and XPF-induced DNA repair	Tirapazamine (TPZ) + glucose oxidase (GOx)	Increased efficacy of TPZ-induced XPF inhibition *via* further increased intratumoral hypoxia	**12-day posttreatment tumor inhibition rate (PDX)** Saline – 0% Cisplatin – 28% Cisplatin + TPZ – 20% GOx + TPZ – 61.72% Cisplatin + GOx + TPZ – 82.08% **25-day posttreatment number of liver metastasis sites per mouse (PDX)** Saline – 100 sites Cisplatin – 100 sites Cisplatin + TPZ – 75 sites GOx + TPZ – 20 sites Cisplatin + GOx + TPZ – 2 sites	[95]
Non-Hodgkin B-cell lymphoma	Cytarabine (antimetabolite)	Activation of G2/M checkpoint	Adavosertib (Wee1 inhibitor)	Inhibition of the acquired activation of G2/M checkpoint	**Number of days until 50% population survival (**in vivo**)** Vehicle – 27 days Cytarabine – 35 days Adavosertib – 25 days Combo – more than 200 days	[68]
	Rituximab (monoclonal antibodies)	Upregulation of inhibitor of apoptosis signaling pathway	LCL161 (SMAC mimetic) + Vinorelbine (Vinca alkaloids)	Inhibition of acquired upregulation of inhibitor of apoptosis signaling pathway	**4-month survival percentage of rituximab-resistant xenograft (**in vivo**)** Control – 15% LCL-161–0% Vinorelbine – 5% Combo – 55%	[49]
Chronic lymphocytic leukemia	Ibrutinib (Bruton’s tyrosine kinase inhibitor)	Increase TCA cycle-induced NADPH production	Etomoxir (CPT1 inhibitor)	Decreased substrate input of TCA cycle	**Survival fraction of 2-day treatment of postibrutinib resistant cancer (**in vitro**)** No treatment – 100% Ibrutinib – 85% Etomoxir – 97% Combo – 45%	[103]
Ovarian cancer	Olaparib (PARP inhibitor)	Upregulation of ATR/CHK1 signaling pathway	Ceralasertib (ATR inhibitor)	Inhibition of the acquired upregulation of ATR/CHK1 signaling pathway	**Weeks until 50% overall survival (PDX)** Control – 6.5 weeks PARP inhibitor – 9 weeks ATR inhibitor – 11 weeks Combo – 25 weeks	[96]
Prostate cancer	Docetaxel (antimicrotubule agent)	Upregulation of mevalonate pathway/YAP axis modulation pathway	Valproic acid (Histone deacetylase inhibitor) + Simvastatin (HMG-CoA reductase inhibitor)	Inhibition of the acquired upregulation of mevalonate pathway/YAP axis modulation pathway	**35-day treatment tumor volume (**in vivo**)** Control – 14.5 cm² Docetaxel – 9 cm² Valproic acid + Simvastatin – 6 cm² Valproic acid + Simvastatin + Docetaxel – 2 cm²	[43]
	Taxanes	Downregulation of CXCR2/Bcl-2 axis	Cisplatin	Further increase the activation of pro-apoptotic pathway	**70-day survival percentage (**in vivo**)** Control – 0% Taxanes – 5% Cisplatin − 0%. Combo – 100% **70-day tumor volume (**in vivo**)** Control − 1.62 cm² Taxanes − 1.4 cm² Cisplatin 1.56 cm² Combo − 0.79 cm²	[35]
	Palbociclib	Upregulation of RPL29 synthesis	Oxaliplatin (platinum-based chemotherapy)	Inhibition of acquired upregulation of RPL29 synthesis	**Percentage of 2-month posttreatment resistant clones (**in vitro**)** Vehicle – 100% Palbociclib – 27.1% Oxaliplatin – 38.5% Combo – 0% **Percentage of 30-day posttreatment resistant clones (PDO)** Palbociclib – 81.3% Oxaliplatin – 100% Combo – 50% **Percentage of metastatic clones (PDO)** Palbociclib – 75% Oxaliplatin – 17.85% Combo – 0%	[90]
Intrahepatic cholangiocarcinoma	Gemcitabine (antimetabolites) and cisplatin	Upregulation of inhibitor of apoptosis signaling pathway	LCL-161	Inhibition of the acquired upregulation of inhibitor of apoptosis signaling pathway	**35-day treatment tumor volume (PDO)** Vehicle – 24.5 cm² LCL-161–20 cm² GEM/CIS – 6.5 cm² Combo – 2.75 cm² **55-day treatment tumor volume (PDO)** GEM/CIS – 16 cm² Combo – 4 cm²	[54]
	Palbociclib	Activation of G1-S phase cell cycle pathway	Sapanisertib (dual m-TOR inhibitor)	Inhibition of the acquired activation of G1-S phase cell cycle pathway	**6.5-week treatment survival rate (**in vivo**)** Control – 0% Palbociclib – 60% Sapanisertib – 40% Combo – 100% **6.5-week treatment tumor weight (**in vivo**)** Control – 6.25 g. Palbociclib – 4.25 g. Sapanisertib – 4 g. Combo – 2 g.	[69]

Most acquired vulnerability studies have yet to demonstrate clinical translatability. Clinical trials are essential to evaluate the potential side effects and toxicities of drug combinations and ensure that the benefits outweigh the risks. Despite their perceived low toxicity, acquired vulnerability approaches targeting metabolic and energy reprogramming require clinical validation to confirm their effectiveness in patients. However, certain acquired vulnerability-based therapies have demonstrated clinical utility. For example, the addition of CDK4/6 inhibitors to hormone deprivation therapy in HR + breast cancer, exploiting the CDK4/6 dependency of HR + breast cancer under hormone deprivation, is now the first-line treatment for advanced HR + breast cancer [143].

## Conclusion and future directions

An acquired vulnerability-based approach for discovering drug combinations has revealed numerous cancer adaptations following resistance to primary treatment. These adaptations confer unique vulnerabilities, rendering cancer cells susceptible to specific drug classes, including noncancer drugs. Identifying targetable alterations in CCA resistance and selecting appropriate novel drug combinations from existing drugs could offer a personalized medicine approach that can continuously adapt to individual patient resistance. Clinical trials are needed to establish the effectiveness of acquired vulnerability-based drug combinations compared to current regimens.

Additionally, exploring other categories of acquired vulnerabilities may offer promising avenues for research. For example, targeting epigenetic dysregulation, which controls downstream proteogenomic characteristics in CCA, could be a potential strategy. Abnormal histone 3 lysine 9 trimethylation (H3K9me3) is correlated with WNT signaling pathway upregulation in intrahepatic CCA [144], and selective histone deacetylase inhibitors can target aberrant histone modifications before and after primary treatment [145]. The upregulation of immunogenic peptides such as PD-L1 and MHC class I in intrahepatic CCA cell lines after trametinib exposure [146] suggests that these peptides may be potential targets for immune checkpoint therapies. Additionally, acquired dysregulation of cytokines, cross-regulated with the anti-apoptotic protein Bcl-2 [35], may offer targets for inflammatory pathway-associated treatments. Drug resistance-induced aberrations in the tumor microenvironment may also present targetable vulnerabilities [147, 148].

These innovative categories may require alternative analyses for acquired vulnerability identification, secondary treatment assessment, and mechanistic understanding. For instance, single-cell barcoding for clonal analysis may be suitable for assessing secondary treatments in heterogeneous cancers such as CCA [149, 150]. Moreover, incorporating alternative treatment modalities, such as radiotherapy, into combination regimens, as demonstrated in oral squamous carcinoma and BRCA1-deficient breast cancer [151, 152], may enhance the efficacy of acquired vulnerability-based approaches. The flexibility of this strategy suggests that there is significant development potential.

In the ideal CCA treatment scenario, where acquired vulnerabilities differ even within the same cancer type and drug response, the challenge lies in identifying molecular subtypes and tailoring drug combinations accordingly. Given the anatomical limitations of biopsy sampling in CCA, developing novel biomarkers from circulating CCA cell components [153, 155] could facilitate subtype detection and acquired vulnerability identification, thereby enhancing the clinical accessibility of this approach. Taken together, these strategies may contribute to the successful implementation of acquired vulnerability-based treatment for CCA patients.

Applying acquired vulnerability-based treatment to create drug combinations for CCA has yielded promising results in preclinical models. This approach may offer a novel strategy for developing potent drug combinations, superseding the current practice of selecting secondary treatments based solely on differences in the mechanism of action from primary treatments [156].

## Data Availability

No datasets were generated or analysed during the current study.

## Abbreviations

5: Fu 5-Fluorouracil
ASC: Active symptom control
CCA: Cholangiocarcinoma
CLL: Chronic lymphocytic leukemia
ER: Estrogen receptor
FADD: Fas-associated death domain
FOLFOX: Fluorouracil, oxaliplatin, and leucovorin
GEM/CIS: Gemcitabine and cisplatin
GSH: Glutathione
GSSG: Oxidized glutathione
HER2: Human epidermal growth factor receptor 2
HIF: Hypoxia-inducible factor
HR: Hormone receptor
IAPs: Inhibitors of apoptosis proteins
MAPK: Mitogen-activated protein kinase
NADPH: Nicotinamide adenine dinucleotide phosphate
NSCLC: Non-small cell lung cancer
OS: Overall survival
OXPHOS: Oxidative phosphorylation
PARP: Poly (ADP-ribose) polymerase
PDO: Patient-derived organoid
PDX: Patient-derived xenograft
ROS: Reactive oxygen species
TCA: Tricarboxylic acid
TNF-α: Tumor necrosis factor-α
TRAIL: TRAILTNF-related apoptosis-inducing ligand
UPR: Unfolded protein response

## References

[1] Banales JM, Marin JJG, Lamarca A, et al. <ArticleTitle Language=“En”>Cholangiocarcinoma 2020: the next horizon in mechanisms and management. Nat Rev Gastroenterol Hepatol. 2020;17(9):557–88. 10.1038/s41575-020-0310-z. 32606456 10.1038/s41575-020-0310-z PMC7447603

[2] Forner A, Vidili G, Rengo M, Bujanda L, Ponz-Sarvisé M, Lamarca A. Clinical presentation, diagnosis and staging of cholangiocarcinoma. Liver Int. 2019;39(Suppl 1):98–107. 10.1111/liv.14086. 30831002 10.1111/liv.14086

[3] Lamarca A, Barriuso J, McNamara MG, Valle JW. Molecular targeted therapies: Ready for prime time in biliary tract cancer. J Hepatol. 2020;73(1):170–85. 10.1016/j.jhep.2020.03.007. 32171892 10.1016/j.jhep.2020.03.007

[4] Goyal L, Meric-Bernstam F, Hollebecque A, et al. Futibatinib for FGFR2-Rearranged Intrahepatic Cholangiocarcinoma. N Engl J Med. 2023;388(3):228–39. 10.1056/NEJMoa2206834. 36652354 10.1056/NEJMoa2206834

[5] Du J, Lv X, Zhang Z, Huang Z, Zhang E. Revisiting targeted therapy and immunotherapy for advanced cholangiocarcinoma. Front Immunol. 2023;14:1142690. Published 2023 Mar 1. 10.3389/fimmu.2023.1142690 10.3389/fimmu.2023.1142690 PMC10014562 36936931

[6] Kelley RK, Bridgewater J, Gores GJ, Zhu AX. Systemic therapies for intrahepatic cholangiocarcinoma. J Hepatol. 2020;72(2):353–63. 10.1016/j.jhep.2019.10.009. 31954497 10.1016/j.jhep.2019.10.009

[7] Cillo U, Fondevila C, Donadon M, et al. Surgery for cholangiocarcinoma. Liver Int. 2019;39(1):143–55. 10.1111/liv.14089. 30843343 10.1111/liv.14089 PMC6563077

[8] Ioka T, Kanai M, Kobayashi S, et al. Randomized phase III study of gemcitabine, cisplatin plus S-1 versus gemcitabine, cisplatin for advanced biliary tract cancer (KHBO1401- MITSUBA). J Hepatobiliary Pancreat Sci. 2023;30(1):102–10. 10.1002/jhbp.1219. 35900311 10.1002/jhbp.1219 PMC10086809

[9] Shroff RT, Javle MM, Xiao L, et al. Gemcitabine, Cisplatin, and nab-Paclitaxel for the Treatment of Advanced Biliary Tract Cancers: A Phase 2 Clinical Trial. JAMA Oncol. 2019;5(6):824–30. 10.1001/jamaoncol.2019.0270. 30998813 10.1001/jamaoncol.2019.0270 PMC6567834

[10] Oh DY, Ruth He A, Qin S, et al. Durvalumab plus Gemcitabine and Cisplatin in Advanced Biliary Tract Cancer. NEJM Evid. 2022;1(8):EVIDoa2200015. 10.1056/EVIDoa2200015. 38319896 10.1056/EVIDoa2200015

[11] Kelley RK, Ueno M, Yoo C et al. Pembrolizumab in combination with gemcitabine and cisplatin compared with gemcitabine and cisplatin alone for patients with advanced biliary tract cancer (KEYNOTE-966): a randomised, double-blind, placebo-controlled, phase 3 trial [published correction appears in Lancet. 2023;402(10406):964] [published correction appears in Lancet. 2024;403(10432):1140]. Lancet. 2023;401(10391):1853–1865. 10.1016/S0140-6736(23)00727-4 10.1016/S0140-6736(23)00727-4 37075781

[12] Lamarca A, Palmer DH, Wasan HS, et al. Second-line FOLFOX chemotherapy versus active symptom control for advanced biliary tract cancer (ABC-06): a phase 3, open-label, randomised, controlled trial. Lancet Oncol. 2021;22(5):690–701. 10.1016/S1470-2045(21)00027-9. 33798493 10.1016/S1470-2045(21)00027-9 PMC8082275

[13] Primrose JN, Fox RP, Palmer DH, et al. Capecitabine compared with observation in resected biliary tract cancer (BILCAP): a randomised, controlled, multicentre, phase 3 study [published correction appears in Lancet Oncol. 2019]. Lancet Oncol. 2019;20(5):663–73. 10.1016/S1470-2045(18)30915-X. 30922733 10.1016/S1470-2045(18)30915-X

[14] Bridgewater J, Fletcher P, Palmer DH, et al. Long-Term Outcomes and Exploratory Analyses of the Randomized Phase III BILCAP Study. J Clin Oncol. 2022;40(18):2048–57. 10.1200/JCO.21.02568. 35316080 10.1200/JCO.21.02568

[15] Wang SJ, Lemieux A, Kalpathy-Cramer J, et al. Nomogram for predicting the benefit of adjuvant chemoradiotherapy for resected gallbladder cancer. J Clin Oncol. 2011;29(35):4627–32. 10.1200/JCO.2010.33.8020. 22067404 10.1200/JCO.2010.33.8020 PMC3236647

[16] Wang Y, Li J, Xia Y, et al. Prognostic nomogram for intrahepatic cholangiocarcinoma after partial hepatectomy. J Clin Oncol. 2013;31(9):1188–95. 10.1200/JCO.2012.41.5984. 23358969 10.1200/JCO.2012.41.5984

[17] Nara S, Esaki M, Ban D, et al. Adjuvant and neoadjuvant therapy for biliary tract cancer: a review of clinical trials. Jpn J Clin Oncol. 2020;50(12):1353–63. 10.1093/jjco/hyaa170. 33037430 10.1093/jjco/hyaa170

[18] Rizzo A, Brandi G. Adjuvant systemic treatment in resected biliary tract cancer: State of the art, controversies, and future directions. Cancer Treat Res Commun. 2021;27:100334. 10.1016/j.ctarc.2021.100334. 33592563 10.1016/j.ctarc.2021.100334

[19] Storandt MH, Jin Z, Mahipal A. Pemigatinib in cholangiocarcinoma with a FGFR2 rearrangement or fusion. Expert Rev Anticancer Ther. 2022;22(12):1265–74. 10.1080/14737140.2022.2150168. 36408971 10.1080/14737140.2022.2150168

[20] Grassian AR, Pagliarini R, Chiang DY. Mutations of isocitrate dehydrogenase 1 and 2 in intrahepatic cholangiocarcinoma. Curr Opin Gastroenterol. 2014;30(3):295–302. 10.1097/MOG.0000000000000050. 24569570 10.1097/MOG.0000000000000050

[21] Abou-Alfa GK, Sahai V, Hollebecque A, et al. Pemigatinib for previously treated, locally advanced or metastatic cholangiocarcinoma: a multicentre, open-label, phase 2 study. Lancet Oncol. 2020;21(5):671–84. 10.1016/S1470-2045(20)30109-1. 32203698 10.1016/S1470-2045(20)30109-1 PMC8461541

[22] Abou-Alfa GK, Macarulla T, Javle MM, et al. Ivosidenib in IDH1-mutant, chemotherapy-refractory cholangiocarcinoma (ClarIDHy): a multicentre, randomised, double-blind, placebo-controlled, phase 3 study [published correction appears in Lancet Oncol. 2020;21(10):e462]. Lancet Oncol. 2020;21(6):796–807. 10.1016/S1470-2045(20)30157-1. 32416072 10.1016/S1470-2045(20)30157-1 PMC7523268

[23] Guo Y, Feng K, Liu Y, et al. Phase I Study of Chimeric Antigen Receptor-Modified T Cells in Patients with EGFR-Positive Advanced Biliary Tract Cancers. Clin Cancer Res. 2018;24(6):1277–86. 10.1158/1078-0432.CCR-17-0432. 29138340 10.1158/1078-0432.CCR-17-0432

[24] Piha-Paul SA, Oh DY, Ueno M, et al. Efficacy and safety of pembrolizumab for the treatment of advanced biliary cancer: Results from the KEYNOTE-158 and KEYNOTE-028 studies. Int J Cancer. 2020;147(8):2190–8. 10.1002/ijc.33013. 32359091 10.1002/ijc.33013

[25] Kim RD, Chung V, Alese OB, et al. A Phase 2 Multi-institutional Study of Nivolumab for Patients With Advanced Refractory Biliary Tract Cancer. JAMA Oncol. 2020;6(6):888–94. 10.1001/jamaoncol.2020.0930. 32352498 10.1001/jamaoncol.2020.0930 PMC7193528

[26] Yoo C, Oh DY, Choi HJ, et al. Phase I study of bintrafusp alfa, a bifunctional fusion protein targeting TGF-β and PD-L1, in patients with pretreated biliary tract cancer. J Immunother Cancer. 2020;8(1):e000564. 10.1136/jitc-2020-000564. 32461347 10.1136/jitc-2020-000564 PMC7254161

[27] Law LW. Resistance in Leukemic Cells to a Guanine Analog, 8-Azaguanine. Proceedings of the Society for Experimental Biology and Medicine. 1951;78(2):499–502. 10.3181/00379727-78-19118 10.3181/00379727-78-19118 14911930

[28] SZYBALSKI W. Genetic studies on microbial cross resistance to toxic agents. I. Cross resistance of Escherichia coli to fifteen antibiotics. J Bacteriol. 1952;64(4):489–99. 10.1128/jb.64.4.489-499.1952. 12999676 10.1128/jb.64.4.489-499.1952 PMC169383

[29] LAMPKIN-HIBBARD JM, MUKHERJEE KL. Effect of steroids and fluoropyrimidines on lymphomas. II. In vivo studies on tumor resistance and collateral sensitivity. Cancer Res. 1963;23:468–76. 13928342

[30] Wang L, Bernards R. Taking advantage of drug resistance, a new approach in the war on cancer. Front Med. 2018;12(4):490–5. 10.1007/s11684-018-0647-7. 30022460 10.1007/s11684-018-0647-7

[31] Piggott L, Silva A, Robinson T, et al. Acquired Resistance of ER-Positive Breast Cancer to Endocrine Treatment Confers an Adaptive Sensitivity to TRAIL through Posttranslational Downregulation of c-FLIP. Clin Cancer Res. 2018;24(10):2452–63. 10.1158/1078-0432.CCR-17-1381. 29363524 10.1158/1078-0432.CCR-17-1381

[32] Schiff R, Reddy P, Ahotupa M, et al. Oxidative stress and AP-1 activity in tamoxifen-resistant breast tumors in vivo. J Natl Cancer Inst. 2000;92(23):1926–34. 10.1093/jnci/92.23.1926. 11106684 10.1093/jnci/92.23.1926

[33] Bergelson S, Pinkus R, Daniel V. Induction of AP-1 (Fos/Jun) by chemical agents mediates activation of glutathione S-transferase and quinone reductase gene expression. Oncogene. 1994;9(2):565–71. 8290267

[34] Pimentel JM, Zhou JY, Wu GS. The Role of TRAIL in Apoptosis and Immunosurveillance in Cancer. Cancers (Basel). 2023;15(10):2752. 10.3390/cancers15102752. Published 2023 May 13. 37345089 10.3390/cancers15102752 PMC10216286

[35] Ruiz de Porras V, Wang XC, Palomero L, et al. Taxane-induced Attenuation of the CXCR2/BCL-2 Axis Sensitizes Prostate Cancer to Platinum-based Treatment. Eur Urol. 2021;79(6):722–33. 10.1016/j.eururo.2020.10.001. 33153817 10.1016/j.eururo.2020.10.001 PMC8088452

[36] Qian S, Wei Z, Yang W, Huang J, Yang Y, Wang J. The role of BCL-2 family proteins in regulating apoptosis and cancer therapy. Front Oncol. 2022;12:985363. Published 2022 Oct 12. 10.3389/fonc.2022.985363 10.3389/fonc.2022.985363 PMC9597512 36313628

[37] Zhou W, Su Y, Zhang Y, Han B, Liu H, Wang X. Endothelial Cells Promote Docetaxel Resistance of Prostate Cancer Cells by Inducing ERG Expression and Activating Akt/mTOR Signaling Pathway. Front Oncol. 2020;10:584505. 10.3389/fonc.2020.584505. Published 2020 Dec 16. 33425737 10.3389/fonc.2020.584505 PMC7793734

[38] Dasari S, Tchounwou PB. Cisplatin in cancer therapy: molecular mechanisms of action. Eur J Pharmacol. 2014;740:364–78. 10.1016/j.ejphar.2014.07.025. 25058905 10.1016/j.ejphar.2014.07.025 PMC4146684

[39] Darshan MS, Loftus MS, Thadani-Mulero M, et al. Taxane-induced blockade to nuclear accumulation of the androgen receptor predicts clinical responses in metastatic prostate cancer. Cancer Res. 2011;71(18):6019–29. 10.1158/0008-5472.CAN-11-1417. 21799031 10.1158/0008-5472.CAN-11-1417 PMC3354631

[40] Hsiao JJ, Ng BH, Smits MM, et al. Androgen receptor and chemokine receptors 4 and 7 form a signaling axis to regulate CXCL12-dependent cellular motility. BMC Cancer. 2015;15:204. 10.1186/s12885-015-1201-5. Published 2015 Mar 31. 25884570 10.1186/s12885-015-1201-5 PMC4393632

[41] Sobolik T, Su YJ, Wells S, Ayers GD, Cook RS, Richmond A. CXCR4 drives the metastatic phenotype in breast cancer through induction of CXCR2 and activation of MEK and PI3K pathways. Mol Biol Cell. 2014;25(5):566–82. 10.1091/mbc.E13-07-0360. 24403602 10.1091/mbc.E13-07-0360 PMC3937084

[42] Wilson C, Purcell C, Seaton A, et al. Chemotherapy-induced CXC-chemokine/CXC-chemokine receptor signaling in metastatic prostate cancer cells confers resistance to oxaliplatin through potentiation of nuclear factor-kappaB transcription and evasion of apoptosis. J Pharmacol Exp Ther. 2008;327(3):746–59. 10.1124/jpet.108.143826. 18780829 10.1124/jpet.108.143826

[43] Iannelli F, Roca MS, Lombardi R et al. Synergistic antitumor interaction of valproic acid and simvastatin sensitizes prostate cancer to docetaxel by targeting CSCs compartment via YAP inhibition. J Exp Clin Cancer Res. 2020;39(1):213. Published 2020 Oct 8. 10.1186/s13046-020-01723-7 10.1186/s13046-020-01723-7 PMC7545949 33032653

[44] Zhang X, Abdelrahman A, Vollmar B, Zechner D. The Ambivalent Function of YAP in Apoptosis and Cancer. Int J Mol Sci. 2018;19(12):3770. 10.3390/ijms19123770. Published 2018 Nov 27. 30486435 10.3390/ijms19123770 PMC6321280

[45] Hatano K, Yamaguchi S, Nimura K, et al. Residual prostate cancer cells after docetaxel therapy increase the tumorigenic potential via constitutive signaling of CXCR4, ERK1/2 and c-Myc. Mol Cancer Res. 2013;11(9):1088–100. 10.1158/1541-7786.MCR-13-0029-T. 23788635 10.1158/1541-7786.MCR-13-0029-T

[46] Fan Y, Hou T, Dan W, et al. ERK1/2 inhibits Cullin 3/SPOP-mediated PrLZ ubiquitination and degradation to modulate prostate cancer progression. Cell Death Differ. 2022;29(8):1611–24. 10.1038/s41418-022-00951-y. 35194188 10.1038/s41418-022-00951-y PMC9345960

[47] Zeng J, Liu W, Fan YZ, He DL, Li L. PrLZ increases prostate cancer docetaxel resistance by inhibiting LKB1/AMPK-mediated autophagy. Theranostics. 2018;8(1):109–123. Published 2018 Jan 1. 10.7150/thno.20356 10.7150/thno.20356 PMC5743463 29290796

[48] Fulda S, Vucic D. Targeting IAP proteins for therapeutic intervention in cancer [published correction appears in Nat Rev Drug Discov. 2012;11(4):331]. Nat Rev Drug Discov. 2012;11(2):109–124. Published 2012 Feb 1. 10.1038/nrd3627 10.1038/nrd3627 22293567

[49] Runckel K, Barth MJ, Mavis C, Gu JJ, Hernandez-Ilizaliturri FJ. The SMAC mimetic LCL-161 displays antitumor activity in preclinical models of rituximab-resistant B-cell lymphoma. Blood Adv. 2018;2(23):3516–25. 10.1182/bloodadvances.2018018168. 30530779 10.1182/bloodadvances.2018018168 PMC6290104

[50] Alas S, Bonavida B. Rituximab inactivates signal transducer and activation of transcription 3 (STAT3) activity in B-non-Hodgkin’s lymphoma through inhibition of the interleukin 10 autocrine/paracrine loop and results in down-regulation of Bcl-2 and sensitization to cytotoxic drugs. Cancer Res. 2001;61(13):5137–44. 11431352

[51] Olejniczak SH, Hernandez FJ, Czuczman MS. Acquirement of rituximab resistance is associated with the development of chemotherapy resistance in B-cell lymphoma cells: Evidence of shared pathways of resistance between chemotherapeutic agents and biological therapies. Blood. 2004;104(11):2297–2297. 10.1182/blood.v104.11.2297.2297.

[52] Yue X, Chen Q, He J. Combination strategies to overcome resistance to the BCL2 inhibitor venetoclax in hematologic malignancies. Cancer Cell Int. 2020;20(1):524. 10.1186/s12935-020-01614-z. Published 2020 Oct 29. 33292251 10.1186/s12935-020-01614-z PMC7597043

[53] Freeze R, Yang KW, Haystead T, Hughes P, Scarneo S. Delineation of the distinct inflammatory signaling roles of TAK1 and JAK1/3 in the CIA model of rheumatoid arthritis. Pharmacol Res Perspect. 2023;11(4):e01124. 10.1002/prp2.1124. 37564034 10.1002/prp2.1124 PMC10415874

[54] Prasopporn S, Suppramote O, Ponvilawan B, et al. Combining the SMAC mimetic LCL-161 with Gemcitabine plus Cisplatin therapy inhibits and prevents the emergence of multidrug resistance in cholangiocarcinoma. Front Oncol. 2022;12:1021632. 10.3389/fonc.2022.1021632. Published 2022 Nov 30. 36531039 10.3389/fonc.2022.1021632 PMC9748615

[55] Chang Y-C, Cheung CH. An updated review of SMAC mimetics, LCL161, Birinapant, and GDC-0152 in cancer treatment. Appl Sci. 2020;11(1):335. 10.3390/app11010335.

[56] Marivin A, Berthelet J, Plenchette S, Dubrez L. The Inhibitor of Apoptosis (IAPs) in Adaptive Response to Cellular Stress. Cells. 2012;1(4):711–37. 10.3390/cells1040711. Published 2012 Oct 10. 24710527 10.3390/cells1040711 PMC3901146

[57] Jin HS, Lee DH, Kim DH, Chung JH, Lee SJ, Lee TH. cIAP1, cIAP2, and XIAP act cooperatively via nonredundant pathways to regulate genotoxic stress-induced nuclear factor-kappaB activation. Cancer Res. 2009;69(5):1782–91. 10.1158/0008-5472.CAN-08-2256. 19223549 10.1158/0008-5472.CAN-08-2256

[58] McCool KW, Miyamoto S. DNA damage-dependent NF-κB activation: NEMO turns nuclear signaling inside out. Immunol Rev. 2012;246(1):311–26. 10.1111/j.1600-065X.2012.01101.x. 22435563 10.1111/j.1600-065X.2012.01101.x PMC3311051

[59] Vashisht M, Ge H, John J, et al. TRAF2/3 deficient B cells resist DNA damage-induced apoptosis via NF-κB2/XIAP/cIAP2 axis and IAP antagonist sensitizes mutant lymphomas to chemotherapeutic drugs. Cell Death Dis. 2023;14(9):599. 10.1038/s41419-023-06122-2. Published 2023 Sep 8. 37679334 10.1038/s41419-023-06122-2 PMC10485046

[60] Niederst MJ, Engelman JA. Bypass mechanisms of resistance to receptor tyrosine kinase inhibition in lung cancer. Sci Signal. 2013;6(294):re6. 10.1126/scisignal.2004652. Published 2013 Sep 24. 24065147 10.1126/scisignal.2004652 PMC3876281

[61] O’Brien NA, McDermott MSJ, Conklin D et al. Targeting activated PI3K/mTOR signaling overcomes acquired resistance to CDK4/6-based therapies in preclinical models of hormone receptor-positive breast cancer. Breast Cancer Res. 2020;22(1):89. Published 2020 Aug 14. 10.1186/s13058-020-01320-8 10.1186/s13058-020-01320-8 PMC7427086 32795346

[62] Jeong SJ, Dasgupta A, Jung KJ, et al. PI3K/AKT inhibition induces caspase-dependent apoptosis in HTLV-1-transformed cells. Virology. 2008;370(2):264–72. 10.1016/j.virol.2007.09.003. 17931677 10.1016/j.virol.2007.09.003 PMC2189985

[63] Zhou H, Li XM, Meinkoth J, Pittman RN. Akt regulates cell survival and apoptosis at a postmitochondrial level. J Cell Biol. 2000;151(3):483–94. 10.1083/jcb.151.3.483. 11062251 10.1083/jcb.151.3.483 PMC2185587

[64] Fassl A, Geng Y, Sicinski P. CDK4 and CDK6 kinases: From basic science to cancer therapy. Science. 2022;375(6577):eabc1495. 10.1126/science.abc1495. 35025636 10.1126/science.abc1495 PMC9048628

[65] Pandey K, Park N, Park KS, et al. Combined CDK2 and CDK4/6 Inhibition Overcomes Palbociclib Resistance in Breast Cancer by Enhancing Senescence. Cancers (Basel). 2020;12(12):3566. 10.3390/cancers12123566. Published 2020 Nov 29. 33260316 10.3390/cancers12123566 PMC7768442

[66] Liu P, Wang Z, Wei W. Phosphorylation of Akt at the C-terminal tail triggers Akt activation. Cell Cycle. 2014;13(14):2162–4. 10.4161/cc.29584. 24933731 10.4161/cc.29584 PMC4111671

[67] Nassar KW, Hintzsche JD, Bagby SM, et al. Targeting CDK4/6 Represents a Therapeutic Vulnerability in Acquired BRAF/MEK Inhibitor-Resistant Melanoma. Mol Cancer Ther. 2021;20(10):2049–60. 10.1158/1535-7163.MCT-20-1126. 34376578 10.1158/1535-7163.MCT-20-1126 PMC9768695

[68] Wang X, Chen Z, Mishra AK, et al. Chemotherapy-induced differential cell cycle arrest in B-cell lymphomas affects their sensitivity to Wee1 inhibition. Haematologica. 2018;103(3):466–76. 10.3324/haematol.2017.175992. 29217775 10.3324/haematol.2017.175992 PMC5830367

[69] Song X, Liu X, Wang H, et al. Combined CDK4/6 and Pan-mTOR Inhibition Is Synergistic Against Intrahepatic Cholangiocarcinoma. Clin Cancer Res. 2019;25(1):403–13. 10.1158/1078-0432.CCR-18-0284. 30084835 10.1158/1078-0432.CCR-18-0284 PMC6423983

[70] McCubrey JA, Steelman LS, Chappell WH, et al. Roles of the Raf/MEK/ERK pathway in cell growth, malignant transformation and drug resistance. Biochim Biophys Acta. 2007;1773(8):1263–84. 10.1016/j.bbamcr.2006.10.001. 17126425 10.1016/j.bbamcr.2006.10.001 PMC2696318

[71] Ming Z, Lim SY, Rizos H. Genetic Alterations in the INK4a/ARF Locus: Effects on Melanoma Development and Progression. Biomolecules. 2020;10(10):1447. Published 2020 Oct 15. 10.3390/biom10101447 10.3390/biom10101447 PMC7602651 33076392

[72] di Ghelli Luserna A, Cerchione C, Martinelli G, Simonetti G. A WEE1 family business: regulation of mitosis, cancer progression, and therapeutic target. J Hematol Oncol. 2020;13(1):126. Published 2020 Sep 21. 10.1186/s13045-020-00959-2 10.1186/s13045-020-00959-2 PMC7507691 32958072

[73] Vakifahmetoglu H, Olsson M, Zhivotovsky B. Death through a tragedy: mitotic catastrophe. Cell Death Differ. 2008;15(7):1153–62. 10.1038/cdd.2008.47. 18404154 10.1038/cdd.2008.47

[74] Sazonova EV, Petrichuk SV, Kopeina GS, Zhivotovsky B. A link between mitotic defects and mitotic catastrophe: detection and cell fate. Biol Direct. 2021;16(1):25. 10.1186/s13062-021-00313-7. Published 2021 Dec 9. 34886882 10.1186/s13062-021-00313-7 PMC8656038

[75] Wang J, Wang Q, Cui Y, et al. Knockdown of cyclin D1 inhibits proliferation, induces apoptosis, and attenuates the invasive capacity of human glioblastoma cells. J Neurooncol. 2012;106(3):473–84. 10.1007/s11060-011-0692-4. 21912938 10.1007/s11060-011-0692-4

[76] Xin Q, Ji Q, Zhang Y, et al. Aberrant ROS Served as an Acquired Vulnerability of Cisplatin-Resistant Lung Cancer. Oxid Med Cell Longev. 2022;2022:1112987. 10.1155/2022/1112987. Published 2022 Jun 20. 35770045 10.1155/2022/1112987 PMC9236771

[77] Ju HQ, Lin JF, Tian T, Xie D, Xu RH. NADPH homeostasis in cancer: functions, mechanisms and therapeutic implications. Signal Transduct Target Ther. 2020;5(1):231. 10.1038/s41392-020-00326-0. Published 2020 Oct 7. 33028807 10.1038/s41392-020-00326-0 PMC7542157

[78] Silva MM, Rocha CRR, Kinker GS, Pelegrini AL, Menck CFM. The balance between NRF2/GSH antioxidant mediated pathway and DNA repair modulates cisplatin resistance in lung cancer cells. Sci Rep. 2019;9(1):17639. 10.1038/s41598-019-54065-6. Published 2019 Nov 27. 31776385 10.1038/s41598-019-54065-6 PMC6881285

[79] Mirzaei S, Mohammadi AT, Gholami MH, et al. Nrf2 signaling pathway in cisplatin chemotherapy: Potential involvement in organ protection and chemoresistance. Pharmacol Res. 2021;167:105575. 10.1016/j.phrs.2021.105575. 33771701 10.1016/j.phrs.2021.105575

[80] Zhao YL, Zhao W, Liu M, Liu L, Wang Y. TBHQ-Overview of Multiple Mechanisms against Oxidative Stress for Attenuating Methamphetamine-Induced Neurotoxicity. Oxid Med Cell Longev. 2020;2020:8874304. Published 2020 Nov 27. 10.1155/2020/8874304 10.1155/2020/8874304 PMC7735854 33354283

[81] Wang N, Song L, Xu Y, et al. Loss of Scribble confers cisplatin resistance during NSCLC chemotherapy via Nox2/ROS and Nrf2/PD-L1 signaling. EBioMedicine. 2019;47:65–77. 10.1016/j.ebiom.2019.08.057. 31495720 10.1016/j.ebiom.2019.08.057 PMC6796531

[82] Corazao-Rozas P, Guerreschi P, Jendoubi M, et al. Mitochondrial oxidative stress is the Achille’s heel of melanoma cells resistant to Braf-mutant inhibitor. Oncotarget. 2013;4(11):1986–98. 10.18632/oncotarget.1420. 24161908 10.18632/oncotarget.1420 PMC3875764

[83] Haq R, Shoag J, Andreu-Perez P, et al. Oncogenic BRAF regulates oxidative metabolism via PGC1α and MITF. Cancer Cell. 2013;23(3):302–15. 10.1016/j.ccr.2013.02.003. 23477830 10.1016/j.ccr.2013.02.003 PMC3635826

[84] Wang L, Leite de Oliveira R, Huijberts S, et al. An Acquired Vulnerability of Drug-Resistant Melanoma with Therapeutic Potential. Cell. 2018;173(6):1413–e142514. 10.1016/j.cell.2018.04.012. 29754815 10.1016/j.cell.2018.04.012

[85] Jyotsana N, Ta KT, DelGiorno KE. The Role of Cystine/Glutamate Antiporter SLC7A11/xCT in the Pathophysiology of Cancer. Front Oncol. 2022;12:858462. 10.3389/fonc.2022.858462. Published 2022 Feb 23. 35280777 10.3389/fonc.2022.858462 PMC8904967

[86] Hayes JD, Dinkova-Kostova AT, Tew KD. Oxidative Stress in Cancer. Cancer Cell. 2020;38(2):167–97. 10.1016/j.ccell.2020.06.001. 32649885 10.1016/j.ccell.2020.06.001 PMC7439808

[87] Yang H, Liang SQ, Xu D, et al. HSP90/AXL/eIF4E-regulated unfolded protein response as an acquired vulnerability in drug-resistant KRAS-mutant lung cancer. Oncogenesis. 2019;8(9):45. 10.1038/s41389-019-0158-7. Published 2019 Aug 20. 31431614 10.1038/s41389-019-0158-7 PMC6702198

[88] Madden E, Logue SE, Healy SJ, Manie S, Samali A. The role of the unfolded protein response in cancer progression: From oncogenesis to chemoresistance. Biol Cell. 2019;111(1):1–17. 10.1111/boc.201800050. 30302777 10.1111/boc.201800050

[89] Szegezdi E, Logue SE, Gorman AM, Samali A. Mediators of endoplasmic reticulum stress-induced apoptosis. EMBO Rep. 2006;7(9):880–5. 10.1038/sj.embor.7400779. 16953201 10.1038/sj.embor.7400779 PMC1559676

[90] Suppramote O, Prasopporn S, Aroonpruksakul S, et al. The Acquired Vulnerability Caused by CDK4/6 Inhibition Promotes Drug Synergism Between Oxaliplatin and Palbociclib in Cholangiocarcinoma. Front Oncol. 2022;12:877194. 10.3389/fonc.2022.877194. Published 2022 May 17. 35664774 10.3389/fonc.2022.877194 PMC9157389

[91] Schmidt HB, Jaafar ZA, Wulff BE, et al. Oxaliplatin disrupts nucleolar function through biophysical disintegration. Cell Rep. 2022;41(6):111629. 10.1016/j.celrep.2022.111629. 36351392 10.1016/j.celrep.2022.111629 PMC9749789

[92] Turi Z, Lacey M, Mistrik M, Moudry P. Impaired ribosome biogenesis: mechanisms and relevance to cancer and aging. Aging. 2019;11(8):2512–40. 10.18632/aging.101922. 31026227 10.18632/aging.101922 PMC6520011

[93] Petrova V, Annicchiarico-Petruzzelli M, Melino G, Amelio I. The hypoxic tumour microenvironment. Oncogenesis. 2018;7(1):10. 10.1038/s41389-017-0011-9. Published 2018 Jan 24. 29362402 10.1038/s41389-017-0011-9 PMC5833859

[94] Kim JW, Tchernyshyov I, Semenza GL, Dang CV. HIF-1-mediated expression of pyruvate dehydrogenase kinase: a metabolic switch required for cellular adaptation to hypoxia. Cell Metab. 2006;3(3):177–85. 10.1016/j.cmet.2006.02.002. 16517405 10.1016/j.cmet.2006.02.002

[95] Chen J, Wang X, Yuan Y, et al. Exploiting the acquired vulnerability of cisplatin-resistant tumors with a hypoxia-amplifying DNA repair-inhibiting (HYDRI) nanomedicine. Sci Adv. 2021;7(13):eabc5267. 10.1126/sciadv.abc5267. Published 2021 Mar 26. 33771859 10.1126/sciadv.abc5267 PMC7997498

[96] Kim H, Xu H, George E et al. Combining PARP with ATR inhibition overcomes PARP inhibitor and platinum resistance in ovarian cancer models. Nat Commun. 2020;11(1):3726. Published 2020 Jul 24. 10.1038/s41467-020-17127-2 10.1038/s41467-020-17127-2 PMC7381609 32709856

[97] Narayanaswamy PB, Tkachuk S, Haller H, Dumler I, Kiyan Y. CHK1 and RAD51 activation after DNA damage is regulated via urokinase receptor/TLR4 signaling. Cell Death Dis. 2016;7(9):e2383. 10.1038/cddis.2016.291. Published 2016 Sep 29. 27685627 10.1038/cddis.2016.291 PMC5059885

[98] Lei P, Wang W, Sheldon M, Sun Y, Yao F, Ma L. Role of Glucose Metabolic Reprogramming in Breast Cancer Progression and Drug Resistance. Cancers (Basel). 2023;15(13):3390. 10.3390/cancers15133390. Published 2023 Jun 28. 37444501 10.3390/cancers15133390 PMC10341343

[99] Yoo HC, Han JM. Amino Acid Metabolism in Cancer Drug Resistance. Cells. 2022;11(1):140. Published 2022 Jan 2. 10.3390/cells11010140 10.3390/cells11010140 PMC8750102 35011702

[100] Safrhansova L, Hlozkova K, Starkova J. Targeting amino acid metabolism in cancer. Int Rev Cell Mol Biol. 2022;373:37–79. 10.1016/bs.ircmb.2022.08.001. 36283767 10.1016/bs.ircmb.2022.08.001

[101] Wang Z, Wang Y, Li Z, Xue W, Hu S, Kong X. Lipid metabolism as a target for cancer drug resistance: progress and prospects. Front Pharmacol. 2023;14:1274335. 10.3389/fphar.2023.1274335. Published 2023 Sep 28. 37841917 10.3389/fphar.2023.1274335 PMC10571713

[102] Brown KK, Spinelli JB, Asara JM, Toker A. Adaptive Reprogramming of De Novo Pyrimidine Synthesis Is a Metabolic Vulnerability in Triple-Negative Breast Cancer [published correction appears in Cancer Discov. 2017;7(7):782]. Cancer Discov. 2017;7(4):391–399. 10.1158/2159-8290.CD-16-0611 10.1158/2159-8290.CD-16-0611 PMC5380483 28255083

[103] Galicia-Vázquez G, Aloyz R. Ibrutinib Resistance Is Reduced by an Inhibitor of Fatty Acid Oxidation in Primary CLL Lymphocytes. Front Oncol. 2018;8:411. 10.3389/fonc.2018.00411. Published 2018 Sep 26. 30319974 10.3389/fonc.2018.00411 PMC6168640

[104] Zhao X, Lwin T, Silva A, et al. Unification of de novo and acquired ibrutinib resistance in mantle cell lymphoma. Nat Commun. 2017;8:14920. 10.1038/ncomms14920. Published 2017 Apr 18. 28416797 10.1038/ncomms14920 PMC5399304

[105] Guan J, Huang D, Yakimchuk K, Okret S. p110α Inhibition Overcomes Stromal Cell-Mediated Ibrutinib Resistance in Mantle Cell Lymphoma. Mol Cancer Ther. 2018;17(5):1090–100. 10.1158/1535-7163.MCT-17-0784. 29483220 10.1158/1535-7163.MCT-17-0784

[106] Kapoor I, Li Y, Sharma A, et al. Resistance to BTK inhibition by ibrutinib can be overcome by preventing FOXO3a nuclear export and PI3K/AKT activation in B-cell lymphoid malignancies. Cell Death Dis. 2019;10(12):924. 10.1038/s41419-019-2158-0. Published 2019 Dec 4. 31801949 10.1038/s41419-019-2158-0 PMC6892912

[107] Csibi A, Lee G, Yoon SO, et al. The mTORC1/S6K1 pathway regulates glutamine metabolism through the eIF4B-dependent control of c-Myc translation. Curr Biol. 2014;24(19):2274–80. 10.1016/j.cub.2014.08.007. 25220053 10.1016/j.cub.2014.08.007 PMC4190129

[108] Pike LS, Smift AL, Croteau NJ, Ferrick DA, Wu M. Inhibition of fatty acid oxidation by etomoxir impairs NADPH production and increases reactive oxygen species resulting in ATP depletion and cell death in human glioblastoma cells. Biochim Biophys Acta. 2011;1807(6):726–34. 10.1016/j.bbabio.2010.10.022. 21692241 10.1016/j.bbabio.2010.10.022

[109] Malaney P, Nicosia SV, Davé V. One mouse, one patient paradigm: New avatars of personalized cancer therapy. Cancer Lett. 2014;344(1):1–12. 10.1016/j.canlet.2013.10.010. 24157811 10.1016/j.canlet.2013.10.010 PMC4092874

[110] Driehuis E, Kretzschmar K, Clevers H. Establishment of patient-derived cancer organoids for drug-screening applications [published correction appears in Nat Protoc. 2021;16(12):5739]. Nat Protoc. 2020;15(10):3380–3409. 10.1038/s41596-020-0379-4 10.1038/s41596-020-0379-4 32929210

[111] Jiang X, Oyang L, Peng Q, et al. Organoids: opportunities and challenges of cancer therapy. Front Cell Dev Biol. 2023;11:1232528. 10.3389/fcell.2023.1232528. Published 2023 Jul 27. 37576596 10.3389/fcell.2023.1232528 PMC10413981

[112] Verduin M, Hoeben A, De Ruysscher D, Vooijs M. Patient-Derived Cancer Organoids as Predictors of Treatment Response. Front Oncol. 2021;11:641980. 10.3389/fonc.2021.641980. Published 2021 Mar 18. 33816288 10.3389/fonc.2021.641980 PMC8012903

[113] Leite de Oliveira R, Wang L, Bernards R. With great power comes great vulnerability. Mol Cell Oncol. 2018;5(6):e1509488. 10.1080/23723556.2018.1509488. Published 2018 Oct 9. 30525088 10.1080/23723556.2018.1509488 PMC6276853

[114] Perwitasari O, Bakre A, Tompkins SM, Tripp RA. siRNA Genome Screening Approaches to Therapeutic Drug Repositioning. Pharmaceuticals (Basel). 2013;6(2):124–60. 10.3390/ph6020124. Published 2013 Jan 28. 24275945 10.3390/ph6020124 PMC3816683

[115] Chou TC. Theoretical basis, experimental design, and computerized simulation of synergism and antagonism in drug combination studies [published correction appears in Pharmacol Rev. 2007;59(1):124]. Pharmacol Rev. 2006;58(3):621–681. 10.1124/pr.58.3.10 10.1124/pr.58.3.10 16968952

[116] Ma J, Motsinger-Reif A. Current Methods for Quantifying Drug Synergism. Proteom Bioinform. 2019;1:43–8. 32043089 PMC7010330

[117] Chou TC. The mass-action law based algorithm for cost-effective approach for cancer drug discovery and development. Am J Cancer Res. 2011;1:925–54. 22016837 PMC3196289

[118] Chou TC. Preclinical versus clinical drug combination studies. Leuk Lymphoma. 2008;49(11):2059–80. 10.1080/10428190802353591. 19021049 10.1080/10428190802353591

[119] Grimaldi A, Santini D, Zappavigna S, et al. Antagonistic effects of chloroquine on autophagy occurrence potentiate the anticancer effects of everolimus on renal cancer cells. Cancer Biol Ther. 2015;16(4):567–79. 10.1080/15384047.2015.1018494. 25866016 10.1080/15384047.2015.1018494 PMC4622435

[120] Shanks RH, Rizzieri DA, Flowers JL, Colvin OM, Adams DJ. Preclinical evaluation of gemcitabine combination regimens for application in acute myeloid leukemia. Clin Cancer Res. 2005;11(11):4225–33. 10.1158/1078-0432.CCR-04-2106. 15930361 10.1158/1078-0432.CCR-04-2106

[121] Flis S, Gnyszka A, Splawinski J. HDAC inhibitors, MS275 and SBHA, enhances cytotoxicity induced by oxaliplatin in the colorectal cancer cell lines. Biochem Biophys Res Commun. 2009;387(2):336–41. 10.1016/j.bbrc.2009.07.017. 19596269 10.1016/j.bbrc.2009.07.017

[122] Terranova-Barberio M, Roca MS, Zotti AI, et al. Valproic acid potentiates the anticancer activity of capecitabine in vitro and in vivo in breast cancer models via induction of thymidine phosphorylase expression. Oncotarget. 2016;7(7):7715–31. 10.18632/oncotarget.6802. 26735339 10.18632/oncotarget.6802 PMC4884949

[123] Bruzzese F, Rocco M, Castelli S, Di Gennaro E, Desideri A, Budillon A. Synergistic antitumor effect between vorinostat and topotecan in small cell lung cancer cells is mediated by generation of reactive oxygen species and DNA damage-induced apoptosis. Mol Cancer Ther. 2009;8(11):3075–87. 10.1158/1535-7163.MCT-09-0254. 19887547 10.1158/1535-7163.MCT-09-0254

[124] Kalra J, Warburton C, Fang K, et al. QLT0267, a small molecule inhibitor targeting integrin-linked kinase (ILK), and docetaxel can combine to produce synergistic interactions linked to enhanced cytotoxicity, reductions in P-AKT levels, altered F-actin architecture and improved treatment outcomes in an orthotopic breast cancer model. Breast Cancer Res. 2009;11(3):R25. 10.1186/bcr2252. 19409087 10.1186/bcr2252 PMC2716491

[125] Cheon J, Lee C, Sang Y, et al. Efficacy and safety of NAB-paclitaxel plus gemcitabine-cisplatin (gemcis/NAB-P) in Korean patients with advanced biliary tract cancers (ABTC): Multicenter Retrospective Analysis. J Clin Oncol. 2021;39(3suppl):274–274. 10.1200/jco.2021.39.3_suppl.274. 10.1177/17588359211035983 PMC8358499 34394748

[126] Jusakul A, Cutcutache I, Yong CH, et al. Whole-Genome and Epigenomic Landscapes of Etiologically Distinct Subtypes of Cholangiocarcinoma. Cancer Discov. 2017;7(10):1116–35. 10.1158/2159-8290.CD-17-0368. 28667006 10.1158/2159-8290.CD-17-0368 PMC5628134

[127] Kongpetch S, Jusakul A, Ong CK, et al. Pathogenesis of cholangiocarcinoma: From genetics to signalling pathways. Best Pract Res Clin Gastroenterol. 2015;29(2):233–44. 10.1016/j.bpg.2015.02.002. 25966424 10.1016/j.bpg.2015.02.002

[128] Thein KZ, Biter AB, Banks KC, et al. Identification of KRASG12C Mutations in Circulating Tumor DNA in Patients With Cancer. JCO Precis Oncol. 2022;6:e2100547. 10.1200/PO.21.00547. 35862868 10.1200/PO.21.00547 PMC9365336

[129] Lee JK, Sivakumar S, Schrock AB, et al. Comprehensive pan-cancer genomic landscape of KRAS altered cancers and real-world outcomes in solid tumors. NPJ Precis Oncol. 2022;6(1):91. 10.1038/s41698-022-00334-z. Published 2022 Dec 9. 36494601 10.1038/s41698-022-00334-z PMC9734185

[130] Loong HH, Du N, Cheng C, et al. KRAS G12C mutations in Asia: a landscape analysis of 11,951 Chinese tumor samples. Transl Lung Cancer Res. 2020;9(5):1759–69. 10.21037/tlcr-20-455. 33209599 10.21037/tlcr-20-455 PMC7653137

[131] Lee J, Park SH, Chang HM et al. Gemcitabine and oxaliplatin with or without erlotinib in advanced biliary-tract cancer: a multicentre, open-label, randomised, phase 3 study [published correction appears in Lancet Oncol. 2012;13(2):e49. Chang, Joung Soon [corrected to Jang, Joung Soon]]. Lancet Oncol. 2012;13(2):181–188. 10.1016/S1470-2045(11)70301-1 10.1016/S1470-2045(11)70301-1 22192731

[132] Rizzo A, Frega G, Ricci AD, et al. Anti-EGFR Monoclonal Antibodies in Advanced Biliary Tract Cancer: A Systematic Review and Meta-analysis. Vivo. 2020;34(2):479–88. 10.21873/invivo.11798. 10.21873/invivo.11798 PMC7157865 32111744

[133] Javle M, Churi C, Kang HC, et al. HER2/neu-directed therapy for biliary tract cancer. J Hematol Oncol. 2015. 10.1186/s13045-015-0155-z. 8:58. Published 2015 May 29. 26022204 10.1186/s13045-015-0155-z PMC4469402

[134] Zhu AX, Meyerhardt JA, Blaszkowsky LS, et al. Efficacy and safety of gemcitabine, oxaliplatin, and bevacizumab in advanced biliary-tract cancers and correlation of changes in 18-fluorodeoxyglucose PET with clinical outcome: a phase 2 study. Lancet Oncol. 2010;11(1):48–54. 10.1016/S1470-2045(09)70333-X. 19932054 10.1016/S1470-2045(09)70333-X

[135] Santoro A, Gebbia V, Pressiani T, et al. A randomized, multicenter, phase II study of vandetanib monotherapy versus vandetanib in combination with gemcitabine versus gemcitabine plus placebo in subjects with advanced biliary tract cancer: the VanGogh study. Ann Oncol. 2015;26(3):542–7. 10.1093/annonc/mdu576. 25538178 10.1093/annonc/mdu576

[136] Bengala C, Bertolini F, Malavasi N, et al. Sorafenib in patients with advanced biliary tract carcinoma: a phase II trial. Br J Cancer. 2010;102(1):68–72. 10.1038/sj.bjc.6605458. 19935794 10.1038/sj.bjc.6605458 PMC2813746

[137] Goyal L, Zheng H, Yurgelun MB, et al. A phase 2 and biomarker study of cabozantinib in patients with advanced cholangiocarcinoma. Cancer. 2017;123(11):1979–88. 10.1002/cncr.30571. 28192597 10.1002/cncr.30571 PMC5444988

[138] Al Baghdadi T, Halabi S, Garrett-Mayer E, et al. Palbociclib in Patients With Pancreatic and Biliary Cancer With CDKN2A Alterations: Results From the Targeted Agent and Profiling Utilization Registry Study. JCO Precis Oncol. 2019;3:1–8. 10.1200/PO.19.00124. 35100714 10.1200/PO.19.00124

[139] Zhang Y, Ma Z, Li C et al. The genomic landscape of cholangiocarcinoma reveals the disruption of post-transcriptional modifiers. Nat Commun. 2022;13(1):3061. Published 2022 Jun 1. 10.1038/s41467-022-30708-7 10.1038/s41467-022-30708-7 PMC9160072 35650238

[140] Yuan S, Norgard RJ, Stanger BZ. Cellular Plasticity in Cancer. Cancer Discov. 2019;9(7):837–51. 10.1158/2159-8290.CD-19-0015. 30992279 10.1158/2159-8290.CD-19-0015 PMC6606363

[141] Yang F, Hilakivi-Clarke L, Shaha A, et al. Metabolic reprogramming and its clinical implication for liver cancer. Hepatology. 2023;78(5):1602–24. 10.1097/HEP.0000000000000005. 36626639 10.1097/HEP.0000000000000005 PMC10315435

[142] Xu X, Chen Y, Shao S, et al. USP21 deubiquitinates and stabilizes HSP90 and ENO1 to promote aerobic glycolysis and proliferation in cholangiocarcinoma. Int J Biol Sci. 2024;20(4):1492–508. 10.7150/ijbs.90774. Published 2024 Feb 4. 38385089 10.7150/ijbs.90774 PMC10878141

[143] Gennari A, André F, Barrios CH, et al. ESMO Clinical Practice Guideline for the diagnosis, staging and treatment of patients with metastatic breast cancer. Ann Oncol. 2021;32(12):1475–95. 10.1016/j.annonc.2021.09.019. 34678411 10.1016/j.annonc.2021.09.019

[144] Hu S, Wang X, Wang T, et al. Differential enrichment of H3K9me3 in intrahepatic cholangiocarcinoma. BMC Med Genomics. 2022;15(1):185. 10.1186/s12920-022-01338-1. Published 2022 Aug 26. 36028818 10.1186/s12920-022-01338-1 PMC9414128

[145] Li Y, Seto E. HDACs and HDAC Inhibitors in Cancer Development and Therapy. Cold Spring Harb Perspect Med. 2016;6(10):a026831. 10.1101/cshperspect.a026831. Published 2016 Oct 3. 27599530 10.1101/cshperspect.a026831 PMC5046688

[146] Wabitsch S, Tandon M, Ruf B, et al. Anti-PD-1 in Combination With Trametinib Suppresses Tumor Growth and Improves Survival of Intrahepatic Cholangiocarcinoma in Mice. Cell Mol Gastroenterol Hepatol. 2021;12(3):1166–78. 10.1016/j.jcmgh.2021.05.011. 34033968 10.1016/j.jcmgh.2021.05.011 PMC8413239

[147] Vaquero J, Lobe C, Tahraoui S, et al. The IGF2/IR/IGF1R Pathway in Tumor Cells and Myofibroblasts Mediates Resistance to EGFR Inhibition in Cholangiocarcinoma. Clin Cancer Res. 2018;24(17):4282–96. 10.1158/1078-0432.CCR-17-3725. 29716918 10.1158/1078-0432.CCR-17-3725

[148] Manabe T, Bivona TG. Remodeling of the tumor/tumor microenvironment ecosystem during KRAS G12C inhibitor clinical resistance in lung cancer. J Clin Invest. 2022;132(4):e156891. 10.1172/JCI156891. 35166243 10.1172/JCI156891 PMC8843703

[149] Howland KK, Brock A. Cellular barcoding tracks heterogeneous clones through selective pressures and phenotypic transitions. Trends Cancer. 2023;9(7):591–601. 10.1016/j.trecan.2023.03.008. 37105856 10.1016/j.trecan.2023.03.008 PMC10339273

[150] Serrano A, Berthelet J, Naik SH, Merino D. Mastering the use of cellular barcoding to explore cancer heterogeneity. Nat Rev Cancer. 2022;22(11):609–24. 10.1038/s41568-022-00500-2. 35982229 10.1038/s41568-022-00500-2

[151] Ohuchi K, Saga R, Hasegawa K, et al. DNAPKcs phosphorylation specific inhibitor, NU7441, enhances the radiosensitivity of clinically relevant radioresistant oral squamous cell carcinoma cells. Biomed Rep. 2023;18(4):28. 10.3892/br.2023.1610. Published 2023 Feb 24. 36926187 10.3892/br.2023.1610 PMC10011949

[152] Barazas M, Gasparini A, Huang Y, et al. Radiosensitivity Is an Acquired Vulnerability of PARPi-Resistant BRCA1-Deficient Tumors. Cancer Res. 2019;79(3):452–60. 10.1158/0008-5472.CAN-18-2077. 30530501 10.1158/0008-5472.CAN-18-2077 PMC6366562

[153] Rodrigues PM, Vogel A, Arrese M, Balderramo DC, Valle JW, Banales JM. Next-Generation Biomarkers for Cholangiocarcinoma. Cancers (Basel). 2021;13(13):3222. Published 2021 Jun 28. 10.3390/cancers13133222 10.3390/cancers13133222 PMC8269024 34203269

[154] Macias RIR, Banales JM, Sangro B, et al. The search for novel diagnostic and prognostic biomarkers in cholangiocarcinoma. Biochim Biophys Acta Mol Basis Dis. 2018;1864(4 Pt B):1468–77. 10.1016/j.bbadis.2017.08.002. 28782657 10.1016/j.bbadis.2017.08.002

[155] Liu Y, Ao X, Ji G, Zhang Y, Yu W, Wang J. Mechanisms of Action And Clinical Implications of MicroRNAs in the Drug Resistance of Gastric Cancer. Front Oncol. 2021;11:768918. 10.3389/fonc.2021.768918. Published 2021 Nov 29. 34912714 10.3389/fonc.2021.768918 PMC8667691

[156] Palmer AC, Sorger PK. Combination Cancer Therapy Can Confer Benefit via Patient-to-Patient Variability without Drug Additivity or Synergy. Cell. 2017;171(7):1678–e169113. 10.1016/j.cell.2017.11.009. 29245013 10.1016/j.cell.2017.11.009 PMC5741091

